# Synergistic effect between cortical cerebral microinfarcts and brain atrophy on cognitive decline

**DOI:** 10.1093/brain/awaf301

**Published:** 2025-08-12

**Authors:** Jiannan Huang, Wei Ying Tan, Jiangbo Cui, Shin Hui On, Eddie Jun Yi Chong, Christopher Chen, Saima Hilal

**Affiliations:** Saw Swee Hock School of Public Health, National University of Singapore and National University Health System, Singapore 117549, Singapore; Saw Swee Hock School of Public Health, National University of Singapore and National University Health System, Singapore 117549, Singapore; Department of Pharmacology, Yong Loo Lin School of Medicine, National University of Singapore, Singapore 117600, Singapore; Department of Pharmacology, Yong Loo Lin School of Medicine, National University of Singapore, Singapore 117600, Singapore; Memory Aging and Cognition Centre, National University Health System, Singapore 117599, Singapore; Department of Pharmacology, Yong Loo Lin School of Medicine, National University of Singapore, Singapore 117600, Singapore; Memory Aging and Cognition Centre, National University Health System, Singapore 117599, Singapore; Department of Pharmacology, Yong Loo Lin School of Medicine, National University of Singapore, Singapore 117600, Singapore; Memory Aging and Cognition Centre, National University Health System, Singapore 117599, Singapore; Saw Swee Hock School of Public Health, National University of Singapore and National University Health System, Singapore 117549, Singapore; Department of Pharmacology, Yong Loo Lin School of Medicine, National University of Singapore, Singapore 117600, Singapore; Memory Aging and Cognition Centre, National University Health System, Singapore 117599, Singapore

**Keywords:** cerebral small vessel disease, neuroimaging, brain volume, cognitive dysfunction, dementia

## Abstract

Cortical cerebral microinfarcts are associated with brain atrophy in cross-sectional studies, with further investigation using longitudinal datasets being warranted. Moreover, little is known about their combined impact on cognition. This study aimed to establish the association between cortical cerebral microinfarcts and brain volume loss over time and explore whether they synergistically contribute to cognitive decline.

A total of 475 patients, aged 72.7 ± 7.9 years, were enrolled from a memory clinic cohort, who underwent neuroimaging and neuropsychological assessments at least twice over 5 years. Cortical cerebral microinfarcts and other cerebrovascular disease were assessed using 3-T MRI. Brain volumes were calculated semi-automatically using FreeSurfer. Cognitive function was assessed using a neuropsychological test battery including six domains. Linear mixed-effect models were utilized to examine the association between cortical cerebral microinfarcts and brain volume loss and their interaction on cognitive decline. Estimated marginal means were derived to plot global cognitive trajectories.

Cortical cerebral microinfarcts were associated with a greater decrease over 2 years in total brain volume [*β* = −1.94 (−3.07, −0.82) at Year 2, *P*-interaction with time < 0.001], grey matter volume [*β* = −1.00 (−1.69, −0.30) at Year 2, *P*-interaction = 0.002] and white matter volume [*β* = −0.95 (−1.54, −0.35) at Year 2, *P*-interaction < 0.001]. Brain volume loss was more pronounced in patients with multiple microinfarcts. Patients with high brain volume loss and cortical cerebral microinfarcts, particularly multiple microinfarcts, exhibited significantly lower global cognitive scores [single microinfarct: *β* = −1.83 (−2.68, −0.97) at Year 5, *P*-interaction with time < 0.001; multiple microinfarcts: *β* = −3.13 (−4.21, −2.05) at Year 5, *P*-interaction < 0.001]. The synergistic effects were more significant in the domains of executive function, memory, language and visuospatial function. Global cognitive trajectories revealed greater cognitive decline in patients with high brain volume loss and single or multiple microinfarcts, with the latter showing the steepest slope.

This study established a longitudinal association between cortical cerebral microinfarcts and brain atrophy progression, with higher microinfarct burden associated with more pronounced brain volume loss. Furthermore, cortical cerebral microinfarcts and brain atrophy showed synergistic effects on cognitive decline. These findings highlight the importance of investigating the role of mixed pathologies in the development of cognitive impairment and dementia in future research.

## Teaser

Huang *et al.* investigate the longitudinal relationship between MRI markers of cerebral small vessel disease and cognitive function. They find that cortical cerebral microinfarcts and brain atrophy interact synergistically to accelerate cognitive decline. These findings highlight the role of mixed pathologies in the development of dementia.

**See Solé-Guardia *et al*. (https://doi.org/10.1093/brain/awaf377) for a scientific commentary on this article.**

## Introduction

Cortical cerebral microinfarcts (CMIs) are small ischaemic lesions (<4 mm in size) restricted to the cerebral cortex, initially detected at brain autopsy and then visualized *in vivo* on structural MRI.^1^ As a novel MRI marker of cerebral small vessel disease (CSVD),^2^ cortical CMIs have been associated with brain functional disorders, including gait disturbances,^3^ neuropsychiatric conditions^4^ and cognitive dysfunction.^5-9^ Although cortical CMIs are common in ageing brains, they are more prevalent in patients with Alzheimer’s disease (AD) and vascular dementia (VaD) compared with those without cognitive impairment.^1^ Moreover, cortical CMIs are associated with a higher incidence of dementia, with an increasing number of lesions correlating with an elevated risk of dementia.^10,11^ Located in the cerebral cortex, the ‘higher-order’ layer critical for cognitive functioning, cortical CMIs may directly contribute to cognitive impairment through their perilesional effects. It has been suggested that cortical CMIs can affect a perilesional zone at least 12-fold larger than the microinfarct core.^12^ Furthermore, cortical CMIs may exert remote effects on brain structural and functional integrity. A previous study found that cortical CMIs were associated with global cortical atrophy, which partially mediated their association with cognitive dysfunction.^13^

Brain atrophy, often regarded as a final common pathway for different pathological processes,^14^ can result from both CSVD and neurodegenerative diseases.^2,15^ Previous studies have reported associations of CSVD markers, including white matter hyperintensities (WMHs),^16-22^ lacunes^21-23^ and perivascular spaces (PVS),^24^ with brain atrophy, both globally and in specific regions like the hippocampus and medial temporal lobe. However, these associations may vary across populations with different aetiologies and stages of cognitive impairment, suggesting complex underlying mechanisms.^19-21,25^ Both cross-sectional^26,27^ and longitudinal studies^10,14^ have shown that brain atrophy is associated with cognitive dysfunction and is an independent risk factor for cognitive decline and dementia conversion. Furthermore, it is suggested that brain atrophy and other CSVD, particularly WMHs, have a combined effect on cognition.^28,29^

Although cortical CMIs have been associated with brain atrophy in cross-sectional studies,^8,13^ longitudinal studies examining their relationship with brain atrophy progression are lacking. Furthermore, it remains unclear whether cortical CMIs and brain atrophy exert combined, additive or synergistic effects on cognitive function. To fill these gaps, this study aimed to: (i) establish the association between cortical CMIs and longitudinal brain volume changes; and (ii) investigate the interaction between cortical CMIs and brain atrophy on cognitive decline over a 5-year follow-up period. We hypothesize that cortical CMIs are associated with a decrease in total brain and/or grey matter volume over time. Furthermore, cortical CMIs and brain atrophy likely interact synergistically to exacerbate cognitive decline.

## Materials and methods

### Study population

The Harmonization study is an ongoing prospective study which recruited patients from memory clinics of the National University Hospital and St. Luke’s Hospital in Singapore, aiming to examine cognitive deterioration and associated biomarkers in older Asian individuals with cognitive impairment and dementia due to AD and/or cerebrovascular disease (CeVD).^8,9^ Patients were eligible and invited to participate if they were: (i) aged 50 years and above; (ii) able to undergo brain MRI; (iii) not diagnosed with aphasia or dysarthria that affected neuropsychological assessments; and (iv) diagnosed with no cognitive impairment, cognitive impairment no dementia and dementia due to AD, VaD or both. Patients with other diagnoses such as Lewy body dementia (LBD) and frontotemporal dementia (FTD) were not included in this cohort. Clinical diagnoses were confirmed during weekly consensus meetings based on well-defined criteria: the National Institute of Neurological and Communicative Disorders and Stroke and the Alzheimer’s Disease and Related Disorders Association (NINCDS-ADRDA) criteria^30^ for AD and the National Institute of Neurological Disorders and Stroke and Association Internationale pour la Recherche et l’ Enseignement en Neurosciences (NINDS-AIREN) criteria^31^ for VaD.

Clinical and neuropsychological assessments were conducted annually from baseline (Year 0) to Year 5 follow-up, while brain MRI scans were performed every 2 years. From August 2010 to February 2020, a total of 700 patients consented to participate and were enrolled in the Harmonization study. Of these, 686 completed a baseline MRI, and 496 had a follow-up MRI at Year 2; furthermore, 634 completed neuropsychological assessments at two or more time points. For the current study, we included 490 patients who had both repeated MRI scans and repeated cognitive assessments. After excluding those with poor image quality (*n* = 9) or missing covariate data (*n* = 6), a total of 475 patients were included in the final analysis. Baseline characteristics of patients in the final analysis and the whole Harmonization cohort are presented in Supplementary Table 1.

This study was approved by the National Healthcare Group Domain-Specific Review Board of Singapore. Informed consent was obtained from all patients. This study was conducted in accordance with the Declaration of Helsinki.

### Neuroimaging

To avoid interscanner variability, all brain MRI scans (baseline and follow-up) were performed using the same whole-body 3T Siemens Magnetom Trio Tim Scanner system with a 32-channel head coil at the Clinical Imaging Research Centre, National University of Singapore. The protocol included T1-weighted images (voxel size = 1 × 1 × 1 mm^3^, repetition time (TR)/echo time (TE)/inversion time (TI) = 2300/1.9/900 ms, flip angle = 9°, field of view (FOV) = 256 × 256 mm^2^), T2-weighted images (voxel size = 1 × 1 × 3 mm^3^, TR/TE = 2600/99 ms, flip angle = 150°, FOV = 232 × 256 mm^2^), Fluid-Attenuated Inversion Recovery (FLAIR) sequences (voxel size = 1 × 1 × 3 mm^3^, TR/TE/TI = 9000/82/2500 ms, flip angle = 180°, FOV = 232 × 256 mm^2^) and susceptibility weighted imaging (SWI) sequences (voxel size = 1 × 1 × 1.5 mm^3^, TR/TE = 27/20 ms, flip angle = 15°, FOV = 192 × 256 mm^2^). The following MRI markers were assessed and included in the data analysis.

#### Cortical CMIs

Cortical CMIs were graded by two raters (J.H., S.H.) independently, based on the Standards for Reporting Vascular Changes on Neuroimaging 2 (STRIVE-2) criteria:^2^ (i) hypointense on T1-weighted images; (ii) hyperintense on T2-weighted images; (iii) hyperintense or isointense (with the surrounding tissue) on FLAIR sequences; (iv) <4 mm in diameter; (v) restricted to the cortex; and (vi) perpendicular to the cortical surface. All identified lesions were discussed in weekly consensus meetings. A subset of 100 images was randomly selected and graded by both raters to calculate inter-rater reliability, which showed good-to-excellent agreement (Cohen’s kappa = 0.88).

#### Brain atrophy

Brain atrophy was assessed by measuring brain volumes from T1-weighted images using FreeSurfer v.6.0 (http://surfer.nmr.mgh.harvard.edu/), according to a well-defined procedure.^32,33^ Briefly, the T1-weighted images were processed using the standard and automated processing stream, including steps such as motion correction, intensity normalization, skull stripping, brain segmentation, tissue classification and volume calculation. To minimize potential segmentation inaccuracies, all segmentations were visually inspected by one investigator (J.H.), and manual adjustments were made when necessary. Grey matter volume (GMV), white matter volume (WMV), total hippocampal volume (THV) and intracranial volume (ICV) were extracted from the FreeSurfer output. Total brain volume (TBV) was calculated as the sum of GMV and WMV. To account for inter-individual variations in head size, TBV, GMV, WMV and THV were normalized to ICV and expressed as percentages (e.g. TBV/ICV × 100%).

#### Other MRI markers

WMHs were segmented using the Lesion Segmentation Toolbox (LST) v.3.0.0 with its lesion growth algorithm, as previously described.^34^ All resulting lesion maps were visually inspected and manually corrected when necessary. WMH volume was then extracted for each patient and normalized to ICV to account for differences in head size (i.e. WMH volume/ICV × 100%). Other CeVD markers were visually graded by one rater (S.H.) blinded to patients’ medical condition, and were treated as binary variables (i.e. present versus absent): (i) cortical infarcts were identified as focal lesions (>5 mm) involving cortical grey matter with a hyperintense rim and a hypointense centre on FLAIR sequences;^15^ (ii) lacunes were identified as hypointense lesions (3–15 mm) involving subcortical regions with a cavity and a hyperintense rim on FLAIR sequences;^15^ (iii) cerebral microbleeds (CMBs) were identified as hypointense lesions (≤10 mm) with a blooming effect on SWI sequences.^35^

### Neuropsychological assessment

The Mini-Mental State Examination (MMSE), Montreal Cognitive Assessment (MoCA) and a formal neuropsychological test battery (National Institute of Neurological Disorders and Stroke and Canadian Stroke Network harmonization battery) were utilized to assess cognitive function. Six cognitive domains were examined in the test battery, which included: attention, executive function, memory, language, visuomotor speed and visuospatial function. The individual tests for each cognitive domain were detailed in our previous studies.^36,37^ Following a previously defined procedure, *Z*-scores for MMSE, MoCA, cognitive domains and global cognition were calculated for data analysis.^36,37^ Briefly, raw scores from individual tests were standardized and then averaged within each cognitive domain to derive domain-specific *Z*-scores. These six domain-specific *Z*-scores were averaged and standardized again to generate global cognitive *Z*-scores.

### Covariates

All patients completed a comprehensive questionnaire documenting demographics such as age, sex, ethnicity, height, weight and years of education. Body mass index (BMI) was calculated as weight/height^2^ (kg/m^2^). Patients’ history of hypertension, hyperlipidaemia, diabetes and atrial fibrillation (AF) was self-reported and verified by medical records and medication use when available. Smoking status was also self-reported and classified as non-smoker or current/ever-smoker.

### Statistical analysis

Patients’ baseline characteristics were summarized as mean ± standard deviation (SD) and median [interquartile range (IQR)] for normally distributed and non-normally distributed continuous variables, respectively, and as count (percentage) for categorical variables. Two-sample *t*-test, Mann–Whitney U-test and Pearson’s chi-square test were employed to compare differences in baseline characteristics between groups.

Baseline cortical CMIs were classified as: (i) absent or present; and (ii) absent, single (=1) or multiple (≥2). Low brain volume loss and high brain volume loss were determined based on the median split of TBV changes from baseline to Year 2. Subsequently, six interaction groups were created: low brain volume loss + CMI absent, low brain volume loss + single CMI, low brain volume loss + multiple CMIs, high brain volume loss + CMI absent, high brain volume loss + single CMI and high brain volume loss + multiple CMIs. Additional analyses were conducted to explore interactions between cortical CMIs and either GMV loss or WMV loss, using the same analytical approach. We also examined the interaction between cortical CMI count and TBV change as continuous variables.

Linear mixed-effect models (‘nlme’ package in R) were utilized to examine the longitudinal association between CMI categories and brain volumetric parameters (TBV, GMV, WMV and THV) from baseline to Year 2, and to investigate the interaction between brain volume loss and CMI categories on cognitive decline across six time points (baseline to Year 5). For both analyses, models with a random intercept or both a random intercept and slope were fitted, based on likelihood ratio tests comparing nested models. Interaction terms between CMI or brain volume loss/CMI categories and time were incorporated and *P*-values for interaction with time were generated. Confounders included age, sex, hypertension, hyperlipidaemia, diabetes, smoking status, AF, cortical infarcts, lacunes, CMBs and WMH volume, with years of education further accounted for when assessing cognitive decline. We also conducted additional analyses including baseline brain volumes or cognitive scores in the models (e.g. baseline TBV in TBV analysis, baseline GMV in GMV analysis, baseline MMSE score in MMSE analysis, baseline MoCA score in MoCA analysis, etc.). Predicted trajectories of brain volumetric parameters and estimated marginal means of global cognitive *Z*-scores were generated using the ‘marginaleffects’ and ‘emmeans’ packages, respectively.

Based on previous literature, large cortical infarcts are strong confounders in the associations of cortical CMIs with other CeVD markers and cognition.^7,8^ Additionally, cortical infarcts may impact brain segmentation when deriving volumetric parameters.^14^ Therefore, we conducted a sensitivity analysis by excluding patients with large cortical infarcts on MRI (*n* = 62), although cortical infarcts have been adjusted for in all regression models. Data analysis was repeated on this subset of patients, as previously described, to assess whether their exclusion influenced our findings.

Statistical analysis was performed using R v.4.2.1 (https://www.r-project.org/), and a *P*-value of <0.05 was considered statistically significant. To account for multiple comparisons across six cognitive domains, Bonferroni correction was applied, and the significance threshold was set at *P*-value < 0.05/6 ≈ 0.008.

## Results

### Baseline characteristics

Demographics, cardiovascular risk factors, MRI markers of CeVD and brain volumetric parameters were compared between patients with and without cortical CMIs (Table 1). Patients with cortical CMIs were more likely to be male and ever/current smokers, had a higher prevalence of hypertension, hyperlipidaemia, diabetes, AF and CeVD, and showed lower TBV, GMV, WMV and THV at both baseline and Year 2, compared with patients without cortical CMIs.

**Table 1:** Baseline characteristics between patients with and without cortical CMIs^a^

Baseline characteristics	Overall (*n* = 475)	CMI absent (*n* = 365)	CMI present (*n* = 110)	*P-* value
Demographics				
Age (years), mean ± SD	72.65 ± 7.86	72.35 ± 7.73	73.66 ± 8.25	0.14
Male, *n* (%)	205 (43.16)	139 (38.08)	66 (60.00)	**<0**.**001**
Body mass index (kg/m^2^), mean ± SD	24.14 ± 3.93	24.09 ± 3.79	24.31 ± 4.35	0.63
Education (years), median (IQR)	6.00 (7.00)	7.00 (7.00)	6.00 (7.00)	0.28
Cardiovascular risk factors				
Ever/current smoker, *n* (%)	114 (24.00)	76 (20.82)	38 (34.55)	**0**.**005**
History of hypertension, *n* (%)	328 (69.05)	243 (66.58)	85 (77.27)	**0**.**044**
History of hyperlipidaemia, *n* (%)	345 (72.63)	252 (69.04)	93 (84.55)	**0**.**002**
History of diabetes, *n* (%)	152 (32.00)	105 (28.77)	47 (42.73)	**0**.**008**
History of atrial fibrillation, *n* (%)	26 (5.47)	12 (3.29)	14 (12.73)	**<0**.**001**
MRI markers of CeVD				
Cortical infarcts, *n* (%)	62 (13.05)	22 (6.03)	40 (36.36)	**<0**.**001**
Lacunes, *n* (%)	123 (25.89)	79 (21.64)	44 (40.00)	**<0**.**001**
Cerebral microbleeds, *n* (%)	208 (43.79)	143 (39.18)	65 (59.09)	**<0**.**001**
WMH volume^b^, median (IQR) (%)	0.34 (0.69)	0.27 (0.59)	0.54 (1.10)	**<0**.**001**
Brain volumetric parameters^b^				
Baseline				
Total brain volume (%), mean ± SD	66.42 ± 5.54	66.99 ± 5.56	64.52 ± 5.08	**<0**.**001**
Grey matter volume (%), mean ± SD	38.48 ± 3.35	38.80 ± 3.34	37.43 ± 3.17	**<0**.**001**
White matter volume (%), mean ± SD	27.93 ± 2.88	28.18 ± 2.86	27.10 ± 2.79	**<0**.**001**
Total hippocampal volume (%), mean ± SD	0.48 ± 0.08	0.48 ± 0.08	0.47 ± 0.07	**0**.**047**
Year 2				
Total brain volume (%), mean ± SD	65.05 ± 6.38	65.89 ± 6.11	62.23 ± 6.48	**<0**.**001**
Grey matter volume (%), mean ± SD	37.64 ± 3.83	38.09 ± 3.65	36.14 ± 4.04	**<0**.**001**
White matter volume (%), mean ± SD	27.40 ± 3.16	27.80 ± 3.05	26.10 ± 3.17	**<0**.**001**
Total hippocampal volume (%), mean ± SD	0.47 ± 0.09	0.47 ± 0.09	0.45 ± 0.08	**0**.**006**

CeVD = cerebrovascular disease; CMI = cerebral microinfarct; IQR = interquartile range; SD = standard deviation; WMH = white matter hyperintensity.

^a^To compare differences in baseline characteristics between patients with and without cortical CMIs, Pearson’s chi-square test, Mann–Whitney U-test and two-sample *t*-test were conducted for categorical, non-normally distributed and normally distributed continuous variables, respectively. The results are denoted in bold if reaching statistical significance.

^b^WMH volume and brain volumetric parameters were normalized to the intracranial volume and reported as a percentage to account for variations in head size.

### Cortical CMIs and brain atrophy

The presence of cortical CMIs was associated with lower TBV, GMV and WMV at Year 2, after adjusting for demographics, cardiovascular risk factors and CeVD markers (Table 2). *P*-interactions between CMI presence and time were significant for these volumetric parameters, indicating accelerated decline over time.

**Table 2:** Association between cortical cerebral microinfarcts and brain volumetric parameters^a^

Categories^b^	Time points	Total brain volume, %		Grey matter volume, %		White matter volume, %		Total hippocampal volume, %	
		*β* (95% CI), *P*-value	*P* ^c^	*β* (95% CI), *P*-value	*P* ^c^	*β* (95% CI), *P*-value	*P* ^c^	*β* (95% CI), *P*-value	*P* ^c^
CMI present	Baseline	−0.74 (−1.87, 0.38), *P* = 0.19	**<0**.**001**	−0.41 (−1.11, 0.28), *P* = 0.24	**0**.**002**	−0.33 (−0.93, 0.27), *P* = 0.28	**<0**.**001**	−0.01 (−0.02, 0.01), *P* = 0.41	**0**.**034**
	Year 2	**−1.94 (−3.07, −0.82), *P* < 0.001**		**−1.00 (−1.69, −0.30), *P* = 0.005**		**−0.95 (−1.54, −0.35), *P* = 0.002**		−0.02 (−0.03, 0.00), *P* = 0.07	
Single CMI	Baseline	−0.40 (−1.80, 1.00), *P* = 0.57	**0**.**014**	−0.36 (−1.23, 0.50), *P* = 0.41	0.14	−0.04 (−0.78, 0.70), *P* = 0.92	**0**.**008**	−0.00 (−0.02, 0.02), *P* = 0.82	0.21
	Year 2	−1.35 (−2.75, 0.05), *P* = 0.06		−0.73 (−1.59, 0.14), *P* = 0.10		−0.62 (−1.36, 0.12), *P* = 0.10		−0.01 (−0.03, 0.01), *P* = 0.41	
Multiple CMIs	Baseline	−1.19 (−2.72, 0.35), *P* = 0.13	**<0**.**001**	−0.49 (−1.43, 0.46), *P* = 0.31	**0**.**001**	−0.70 (−1.51, 0.12), *P* = 0.09	**0**.**005**	−0.01 (−0.04, 0.01), *P* = 0.26	0.05
	Year 2	**−2.68 (−4.21, −1.15), *P* < 0.001**		**−1.32 (−2.27, −0.38), *P* = 0.006**		**−1.36 (−2.17, −0.54), *P* = 0.001**		**−0.02 (−0.05, −0.00), *P* = 0.043**	

CI = confidence interval; CMI = cerebral microinfarct.

^a^Brain volumetric parameters were normalized to the intracranial volume and reported as a percentage to account for variations in head size. Model adjusted for age, sex, hypertension, hyperlipidaemia, diabetes, smoking status, atrial fibrillation, cortical infarcts, lacunes, cerebral microbleeds and white matter hyperintensity volume. The results are denoted in bold if reaching statistical significance.

^b^Cortical CMIs were categorized as: (1) absent and present; (2) absent, single (=1), and multiple (≥2). Patients without cortical CMIs served as the reference group.

^c^ *P*-value for the interaction between CMI category and time.

**Figure 1:**
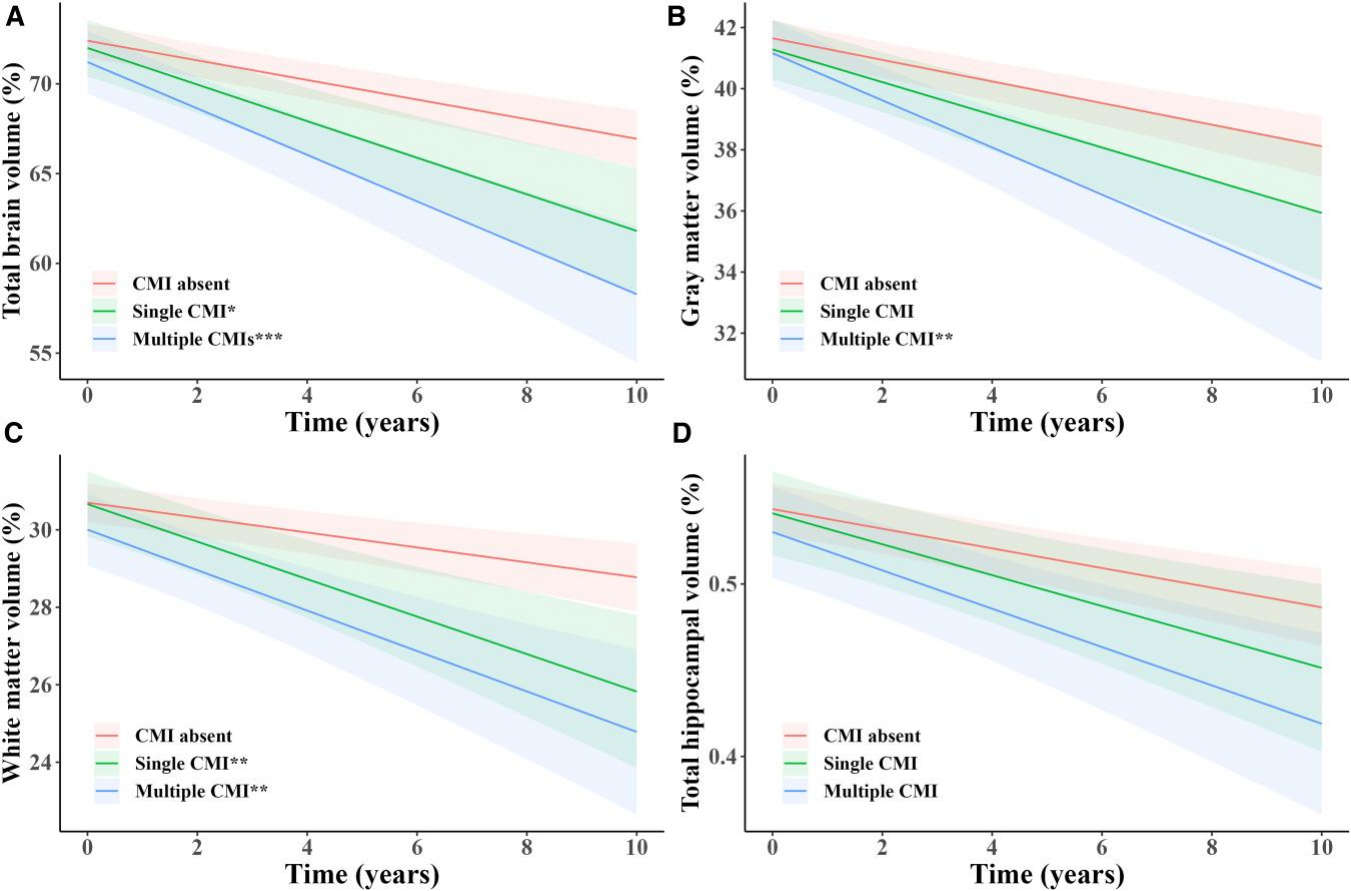
**Predicted brain volume loss over time by cortical cerebral microinfarct categories**. Linear mixed-effect models were used to predict changes over time in total brain volume (**A**), grey matter volume (**B**), white matter volume (**C**) and total hippocampal volume (**D**) based on cortical cerebral microinfarct categories, adjusting for age, sex, hypertension, hyperlipidaemia, diabetes, smoking status, atrial fibrillation, cortical infarcts, lacunes, cerebral microbleeds and white matter hyperintensity volume. *P-interaction with time <0.05, **P-interaction with time <0.01, ***P-interaction with time <0.001. CMI = cerebral microinfarct.

We further categorized CMI presence as single or multiple and found that multiple CMIs were significantly associated with lower TBV, GMV, WMV and THV at Year 2, after adjusting for confounders. Additionally, multiple CMIs exhibited significant interactions with time in relation to brain volume loss. No significant associations were observed between a single CMI and brain volumetric parameters, although the association with TBV at Year 2 approached statistical significance. As shown in Fig. 1, greater reductions in predicted brain volumes were observed with higher cortical CMI burden, with the steepest decline typically associated with multiple CMIs.

After additionally adjusting for baseline brain volumes (Supplementary Table 2), the presence of cortical CMIs remained associated with lower TBV, GMV and WMV at Year 2. Significant associations were also observed between multiple CMIs and TBV, GMV, WMV and THV at Year 2.

### Interaction between cortical CMIs and brain atrophy on cognitive decline

**Figure 2:**
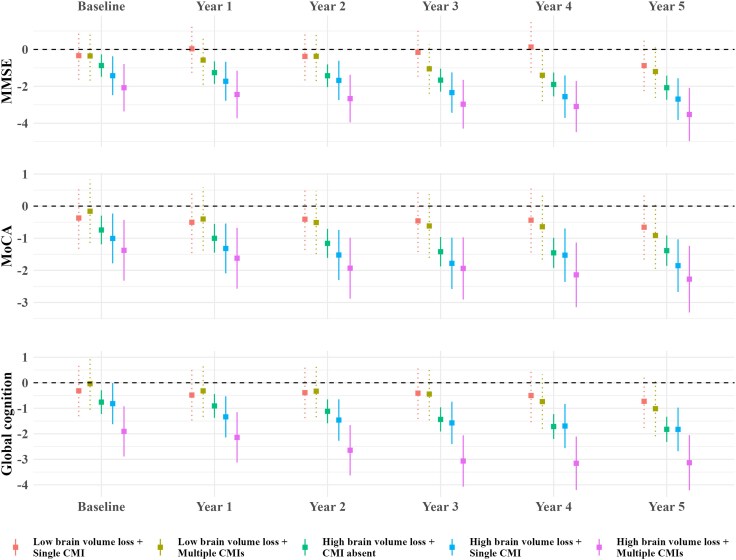
**Synergistic effects of cortical cerebral microinfarcts and brain atrophy on global cognitive function**. Effect estimates from linear mixed-effect models were used to visualize longitudinal associations between brain volume loss/cortical cerebral microinfarct interaction groups and *Z*-scores of the Mini-Mental State Examination, Montreal Cognitive Assessment and global cognition, adjusting for age, sex, years of education, hypertension, hyperlipidaemia, diabetes, smoking status, atrial fibrillation, cortical infarcts, lacunes, cerebral microbleeds and white matter hyperintensity volume. Patients with low brain volume loss and no cortical cerebral microinfarcts served as the reference group. Solid lines denote statistical significance. CMI = cerebral microinfarct; MMSE = Mini-Mental State Examination; MoCA = Montreal Cognitive Assessment.

Compared with patients with low brain volume loss and no cortical CMIs, patients with high brain volume loss and cortical CMIs, particularly multiple CMIs, exhibited significantly lower scores in MMSE, MoCA and global cognition across all time points, after adjusting for demographics, cardiovascular risk factors and CeVD markers (Fig. 2 and Supplementary Table 3). *P*-interactions with time were also significant, indicating accelerated cognitive decline over time.

**Figure 3:**
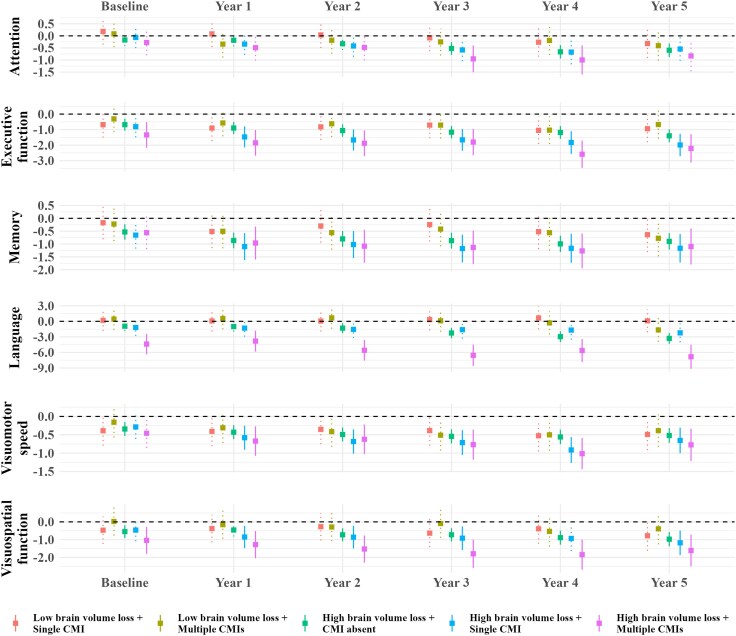
**Synergistic effects of cortical cerebral microinfarcts and brain atrophy on cognitive domains**. Effect estimates from linear mixed-effect models were used to visualize longitudinal associations between brain volume loss/cortical cerebral microinfarct interaction groups and domain-specific cognitive *Z*-scores, adjusting for age, sex, years of education, hypertension, hyperlipidaemia, diabetes, smoking status, atrial fibrillation, cortical infarcts, lacunes, cerebral microbleeds and white matter hyperintensity volume. Patients with low brain volume loss and no cortical cerebral microinfarcts served as the reference group. Solid lines denote statistical significance after applying Bonferroni correction. CMI = cerebral microinfarct.

High brain volume loss was associated with lower scores across all six cognitive domains, with pronounced effects on executive function, memory, language and visuospatial function, even after adjusting for confounders and applying Bonferroni correction (Fig. 3 and Supplementary Table 4). Additionally, in the three high brain volume loss groups, cognitive scores further declined with higher cortical CMI burden and at later time points (e.g. Years 3 to 5). Significant *P*-interactions with time indicated an accelerated decline across all cognitive domains over time.

After additional adjustment for baseline cognitive scores, patients with high brain volume loss and cortical CMIs, particularly multiple CMIs, still showed significantly lower scores in MMSE, MoCA and global cognition, especially at later time points, compared with those with low brain volume loss and no cortical CMIs (Supplementary Fig. 1). Moreover, patients with high brain volume loss and cortical CMIs generally had lower scores in executive function, memory and visuomotor speed (Supplementary Fig. 2).

Furthermore, a significant interaction between cortical CMI count and TBV change (both as continuous variables) was observed for declines in MMSE, MoCA, global cognition, memory and language scores (Supplementary Table 5).

### Global cognitive trajectories by cortical CMI and brain atrophy categories

**Figure 4:**
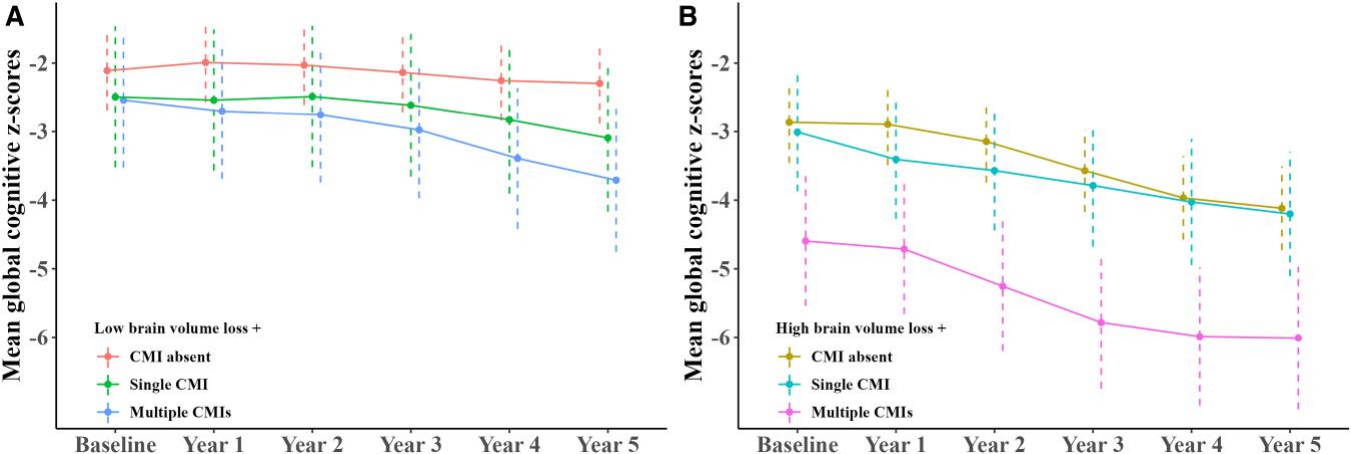
**Global cognitive trajectories by cortical cerebral microinfarct and brain atrophy categories**. The estimated marginal means of global cognitive *Z*-scores at six time points were calculated based on linear mixed-effect models and used to plot global cognitive trajectories for brain volume loss/cortical cerebral microinfarct interaction groups. Trajectories were plotted separately for low (**A**) and high (**B**) brain volume loss groups. CMI = cerebral microinfarct.

The combined effect of cortical CMIs with brain atrophy progression on global cognitive trajectories is shown in Fig. 4. Patients with low brain volume loss exhibited global cognitive decline over time, particularly those with multiple CMIs (Fig. 4A). The same trend was observed in patients with high brain volume loss, who generally had lower global cognitive scores than those with low brain volume loss (Fig. 4B). However, the discrepancy in global cognitive trajectories across CMI categories was more pronounced in the high brain volume loss groups compared with the low brain volume loss groups. Furthermore, patients with high brain volume loss and multiple CMIs exhibited the most pronounced global cognitive decline over time (*P*-interaction with time < 0.001), indicating a synergistic effect between cortical CMIs and brain atrophy progression.

Similar patterns were observed in additional analyses examining the interaction between cortical CMIs and either GMV loss or WMV loss (Supplementary Fig. 3).

### Sensitivity analysis

In patients without cortical infarcts, the presence of cortical CMIs remained associated with lower TBV at Year 2, with significant *P*-interaction indicating accelerated volume loss over time (Supplementary Table 6). Significant associations were also observed between multiple CMIs and TBV, GMV and WMV at Year 2, after adjusting for confounders, with significant *P*-interactions with time. No significant associations were observed between a single CMI and brain volumetric parameters.

The combined effect between cortical CMIs and brain atrophy on global cognitive function remained largely unchanged after excluding cortical infarcts (Supplementary Table 7). Significant associations were found in the three high brain volume loss groups, consistent across all cognitive tests and time points. In addition, lower cognitive scores were observed with higher cortical CMI burden, with significant *P*-interactions over time indicating accelerated cognitive decline. Similarly, high brain volume loss was associated with lower scores across six cognitive domains (Supplementary Table 8). These associations were generally more pronounced with higher cortical CMI burden and at later time points. Significant *P*-interactions with time also indicated accelerated decline over time.

## Discussion

In a memory clinic population, we found that cortical CMIs were associated with greater and accelerated brain volume loss, which was more pronounced with higher cortical CMI burden. Furthermore, higher brain volume loss, combined with multiple cortical CMIs, was associated with greater and accelerated cognitive decline over 5 years. These findings support our hypothesis that cortical CMIs and brain atrophy synergistically contribute to cognitive dysfunction and decline.

The association between cortical CMIs and brain atrophy was initially established in a few autopsy studies.^38,39^ After their visualization on MRI, this association was further confirmed *in vivo*, with findings indicating that cortical CMIs are associated with lower TBV and GMV.^8,10,13^ However, these findings were limited by the cross-sectional design, and the reported associations were either unadjusted or only partially adjusted for demographics and a few CeVD markers. In a memory clinic population with MRI data at two time points, we found that the presence of cortical CMIs was associated with 3.0%, 2.7% and 3.5% reductions in TBV, GMV and WMV, respectively, at the Year 2 follow-up, after adjusting for confounders. Additionally, CMI presence was associated with an accelerated decrease in brain volumes over 2 years. A dose–response relationship was further observed, with higher cortical CMI burden associated with greater and faster brain volume loss. To our knowledge, this study is the first to establish longitudinal associations between cortical CMIs and brain atrophy, providing an important extension to previous research. Notably, the associations between cortical CMIs and brain volume loss in our study remained significant even after adjusting for cardiovascular risk factors and CeVD markers. This suggests that cortical CMIs may independently contribute to brain atrophy, beyond shared aetiologies and risk factors such as hypertension and other forms of CSVD. It is suggested that cortical CMIs may cause perilesional cortical damage in an area up to 150 times greater than the CMI core, involving pathological changes such as ischaemia-related neuronal death, neuroinflammation and blood–brain barrier leakage.^13^ This perilesional effect is supported by previous findings showing lower cortical thickness within a 20 mm radius of the CMI core compared with areas 20–50 mm away, as well as in regions with cortical CMIs compared with contralateral, CMI-free reference regions.^13^ Given that detecting a single cortical CMI reflects the presence of hundreds to thousands of similar lesions throughout the brain,^1^ the cumulative effect of these lesions may further contribute to widespread cortical damage, cortical thinning and ultimately measurable reductions in global GMV. This may explain why cortical CMIs are related to GMV and why a higher cortical CMI burden leads to greater brain volume loss, as evidenced in our study. We also observed a significant association between cortical CMIs and WMV loss. This is consistent with prior research showing that cortical CMIs can lead to white matter damage.^37,40^ Evidence suggests that cortical CMIs may damage adjacent white matter fibres, resulting in demyelination and axonal injury, and may also impair remote white matter regions through secondary degeneration or disruption of long-range fibre pathways.^1,41^ Additionally, cortical CMIs have been associated with glymphatic dysfunction, which could impede the clearance of neurotoxic metabolites and further contribute to white matter damage.^41^ While cortical CMIs can result in perilesional grey matter loss, they could also be linked to broader grey and white matter atrophy, likely via multiple direct and indirect mechanisms as previously discussed. Furthermore, multiple CMIs in our study were associated with hippocampal atrophy, a key biomarker for AD diagnosis and treatment.^16^ Increasing evidence suggests that mixed pathologies, particularly coexisting vascular pathologies, play an important role in the development of AD,^42^ a notion reinforced by our findings. The interplay between cortical CMIs and AD pathologies warrants further investigation.

Brain atrophy and other forms of CSVD are established as independent risk factors for cognitive impairment and dementia.^2^ Several studies have also explored their combined effects, showing that brain atrophy and WMHs can synergistically contribute to cognitive dysfunction. For instance, a hospital-based study demonstrated a significant interaction between WMHs and hippocampal atrophy on memory decline over 8.7 years.^28^ Additionally, a community-based study observed an additive increment in dementia risk over 5 years with higher WMH volumes and more severe brain atrophy.^29^ These findings indicate that cognitive dysfunction in CSVD is a heterogeneous process involving diverse pathologies. Unlike WMHs, cortical CMIs are located in grey matter and thus may exert more direct effects on cognitive function. However, research on the interrelations between cortical CMIs, brain atrophy and cognition remains limited. This study addresses this gap by demonstrating a synergistic effect between cortical CMIs and brain atrophy on cognitive function, with more cortical CMIs and higher brain volume loss associated with greater and faster cognitive decline. The additional analyses focusing on GMV and WMV loss reinforced the robustness of this finding and suggest that both grey and white matter atrophy may reflect overlapping neurodegenerative pathways that compound the effects of cortical CMIs on cognitive deterioration. Additionally, we found that executive function, memory, language and visuospatial function were the most affected cognitive domains, consistent with previous findings.^7,28,43^ The co-occurrence of cortical CMIs and brain atrophy may indicate more severe pathological changes in the brain vasculature, contributing to poorer cognitive performance. Furthermore, although brain atrophy, particularly hippocampal atrophy, may occur in conditions beyond AD, AD pathologies likely play a key role in its development. Thus, the observed synergistic effect may reflect a complex interaction between vascular and AD pathologies. Previous evidence indicates that concomitant vascular and AD pathologies can double the risk of dementia.^11^ While our cohort excluded patients with clinical diagnoses of other neurodegenerative diseases such as LBD and FTD, we acknowledge that these pathologies may still be present at preclinical stages, and their influence cannot be entirely ruled out. It is also possible that patients with both cortical CMIs and brain atrophy have a higher burden of cardiovascular risk factors or CeVD, which may exacerbate cognitive dysfunction. However, these potential contributors are unlikely to explain our findings, as they were adjusted for in the analysis. Future research should focus on elucidating the mechanisms underlying these interactions.

Given the strong association of large cortical infarcts with cortical CMIs, brain atrophy and cognitive impairment, we conducted a sensitivity analysis in a subset of patients without cortical infarcts. The results remained largely unchanged, highlighting the robustness of our findings.

A major strength of this study is its longitudinal design with multiple neuroimaging and neuropsychological assessments over 5 years of follow-up, allowing us to investigate the temporal relationships between cortical CMIs, brain atrophy and cognition. Additionally, cognitive function was assessed using a comprehensive neuropsychological test battery. The consistent results observed across different cognitive tests and domains enhance the reliability of our findings. Moreover, brain atrophy was assessed using auto-generated brain volumetric parameters rather than visual scales, minimizing observer bias.

This study has some limitations that warrant discussion. First, this study was conducted in a memory clinic population, which has a higher prevalence of cardiovascular risk factors and CeVD compared with the general population. Caution should be exercised when generalizing our findings to other populations. Second, cortical CMIs were graded on 3T MRI, which can detect only a subset of larger lesions. It remains unclear whether the observed association between cortical CMIs and brain atrophy extends to smaller CMIs. Due to ongoing data collection, we were unable to examine the relationship between incident cortical CMIs and brain atrophy progression. This analysis may provide additional insight into the dynamic interplay between these two MRI markers and will be addressed in our future research. Third, learning effects in repeated neuropsychological assessments, especially at later time points, may lead to an underestimation of cognitive decline in our study sample. Fourth, attrition bias is inevitable for longitudinal studies. Although the average annual loss to follow-up rate was relatively low (11.7%), patients with better compliance may be healthier and differ from those lost to follow-up in terms of brain atrophy progression and cognitive decline. Fifth, other potential sources of selection bias should also be considered. Some individuals who declined participation or were excluded from the Harmonization study were likely to be functionally dependent, possibly due to advanced dementia or comorbidities. This may limit the generalizability of our findings to patients with poorer health status. Additionally, patients who did not complete MRI scans or had poor compliance resulting in poor image quality were excluded from the analysis. They may have had stents or more severe CeVD, and therefore differ from those who completed high-quality, repeated MRI scans in terms of vascular risk factor burden. Sixth, we were unable to differentiate cortical CMIs of cardio-embolic origin from those due to CSVD pathologies (e.g. arteriolosclerosis), based on their neuroimaging appearance. Although we have adjusted for AF, a key risk factor for cardio-embolism,^1^ cortical CMIs of different aetiologies may have distinct prognostic implications. Finally, while we adjusted for a wide range of confounders, we cannot fully exclude the potential influence of unmeasured confounders on the observed associations.

In conclusion, this study established a longitudinal association between cortical CMIs and brain atrophy progression in a memory clinic population. Moreover, we demonstrated a synergistic effect between cortical CMIs and brain atrophy on cognitive decline. These findings extend our knowledge of the relationships between different forms of CSVD and cognition, underscoring the importance of investigating the role of mixed pathologies, particularly vascular and AD pathologies, in the development of cognitive impairment and dementia in future research.

## Supplementary Material

awaf301_Supplementary_Data

## Data Availability

The data that support the findings of this study are available from the corresponding author upon reasonable request.
