# Supplementary material for: Synergistic effect between cortical cerebral microinfarcts and brain atrophy on cognitive decline
Source: Brain. 2025 Aug 12;148(11):3924–34. doi: 10.1093/brain/awaf301 (PMC12588707; doi:10.1093/brain/awaf301)
Supplement: awaf301_Supplementary_Data [file awaf301_supplementary_data.pdf]

Supplementary Material

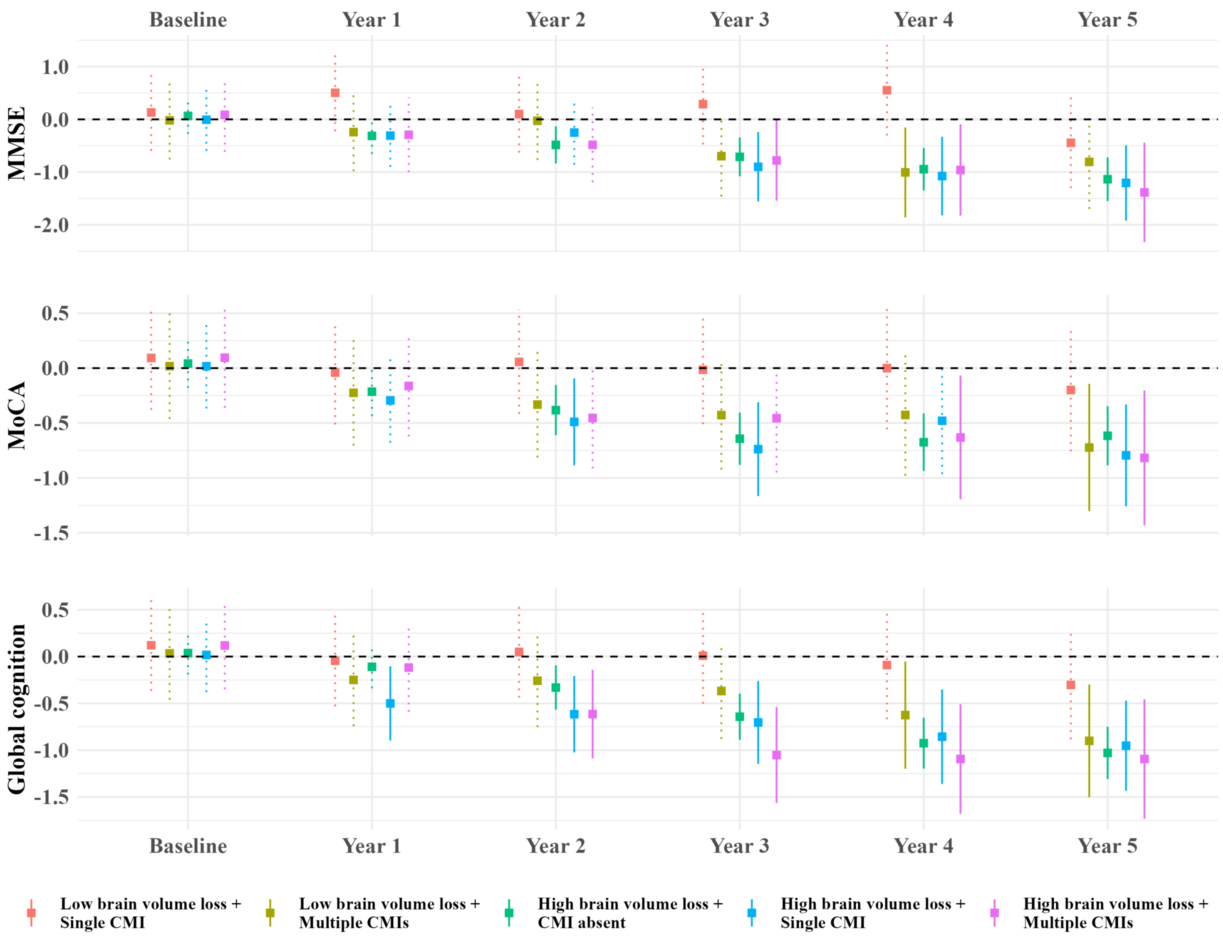

Supplementary Figure 1. Synergistic effects of cortical cerebral microinfarcts and brain atrophy on global cognitive function after adjustment for baseline cognitive scores

Effect estimates from linear mixed-effect models were used to visualize longitudinal associations between brain volume loss/cortical cerebral microinfarct interaction groups and z-scores of the Mini-Mental State Examination, Montreal Cognitive Assessment, and global cognition, adjusting for age, sex, years of education, hypertension, hyperlipidemia, diabetes, smoking status, atrial fibrillation, cortical infarcts, lacunes, cerebral microbleeds, white matter hyperintensity volume, and baseline cognitive scores. Patients with low brain volume loss and no cortical cerebral microinfarcts served as the reference group. Solid lines denote statistical significance.

Abbreviation: CMI, cerebral microinfarct; MMSE, Mini-Mental State Examination; MoCA, Montreal Cognitive Assessment.

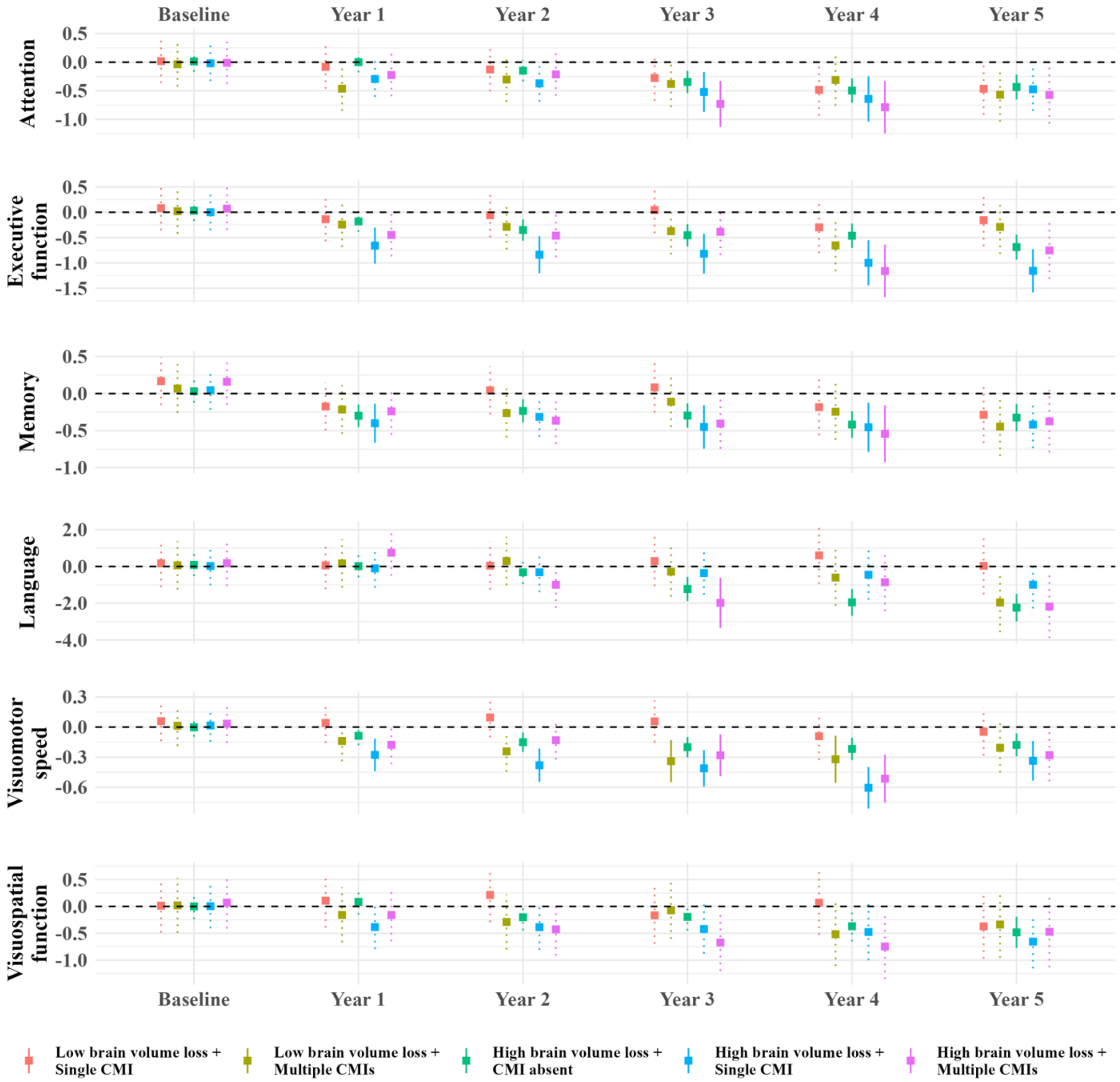

Supplementary Figure 2. Synergistic effects of cortical cerebral microinfarcts and brain atrophy on cognitive domains after adjustment for baseline cognitive scores

Effect estimates from linear mixed-effect models were used to visualize longitudinal associations between brain volume loss/cortical cerebral microinfarct interaction groups and domain-specific cognitive z-scores, adjusting for age, sex, years of education, hypertension, hyperlipidemia, diabetes, smoking status, atrial fibrillation, cortical infarcts, lacunes, cerebral microbleeds, white matter hyperintensity volume, and baseline cognitive scores. Patients with low brain volume loss

and no cortical cerebral microinfarcts served as the reference group. Solid lines denote statistical significance after applying Bonferroni correction.
Abbreviation: CMI, cerebral microinfarct.

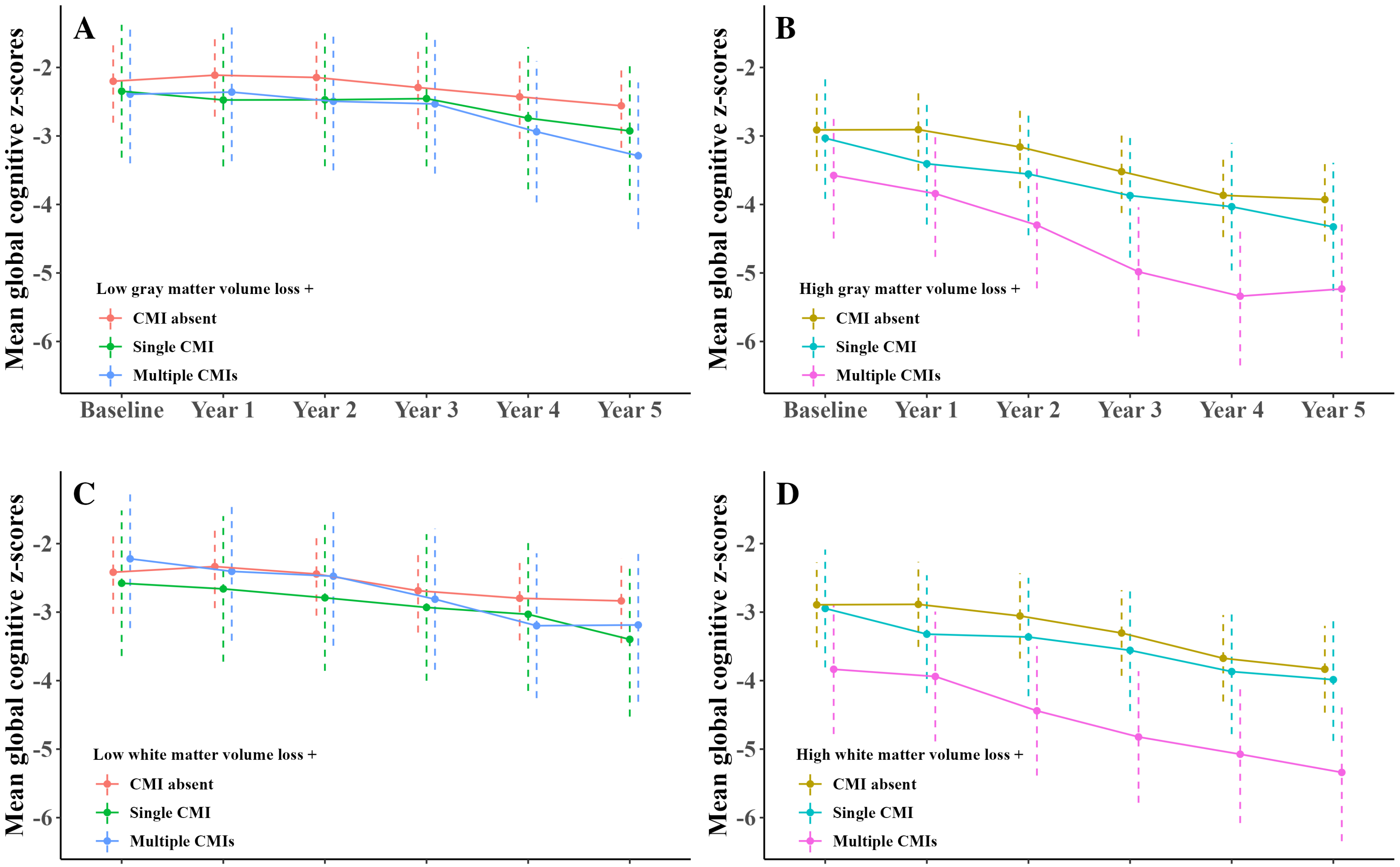
A
B
C
D
Mean global cognitive z-scores
-2
-3
-4
-5
-6
Baseline
Year 1
Year 2
Year 3
Year 4
Year 5
Low gray matter volume loss +
High gray matter volume loss +
Low white matter volume loss +
High white matter volume loss +
CMI absent
Single CMI
Multiple CMIs

Supplementary Figure 3. Interaction between cortical cerebral microinfarcts and gray/white matter volume loss on global cognitive trajectories

The estimated marginal means of global cognitive z-scores at six time points were calculated based on linear mixed-effect models and used to plot global cognitive trajectories across interaction groups between cortical cerebral microinfarcts and either gray matter volume loss (A, B) or white matter volume loss (C, D). Trajectories were plotted separately for low and high gray/white matter volume loss groups.

Abbreviation: CMI, cerebral microinfarct.

Supplementary Table 1. Baseline characteristics of patients included in the current study compared to the whole Harmonization cohort[a]

Baseline characteristics	Harmonization (n=700)	Patients included (n=475)	p value
**Demographics**			
Age, mean (SD), year	72.94 (7.95)	72.65 (7.86)	0.54
Male, n (%)	304 (43.43)	205 (43.16)	0.97
Body mass index, mean (SD), kg/m^2	23.87 (3.99)	24.14 (3.93)	0.26
Education, median (IQR), year	6.00 (7.00)	6.00 (7.00)	0.32
**Cardiovascular risk factors**			
Ever/Current smoker, n (%)	185 (26.43)	114 (24.00)	0.38
History of hypertension, n (%)	489 (69.86)	328 (69.05)	0.66
History of hyperlipidemia, n (%)	498 (71.14)	345 (72.63)	0.61
History of diabetes, n (%)	235 (33.57)	152 (32.00)	0.84
History of atrial fibrillation, n (%)	45 (6.45)	26 (5.47)	0.57
**Clinical diagnosis**			
No cognitive impairment, n (%)	132 (18.86)	105 (22.11)	0.15
Cognitive impairment no dementia, n (%)	290 (41.43)	206 (43.37)	
Dementia, n (%)	278 (39.71)	164 (34.53)	

[a]To compare differences in baseline characteristics between two groups, Pearson's Chi-square test, Mann-Whitney U test, and two-sample t test were conducted for categorical, non-normally distributed, and normally distributed continuous variables, respectively.

Supplementary Table 2. Association between cortical cerebral microinfarcts and brain volumetric parameters adjusted for baseline volumes[a]

Categories[b]	Time points	Total brain volume, %		Gray matter volume, %		White matter volume, %		Total hippocampal volume, %	
		β (95%CI), p value	p[c]	β (95%CI), p value	p[c]	β (95%CI), p value	p[c]	β (95%CI), p value	p[c]
**CMI present**	Baseline	0.06 (-0.38, 0.50), p=0.78	**<0.001**	0.05 (-0.23, 0.33), p=0.74	**0.002**	0.01 (-0.23, 0.26), p=0.91	**<0.001**	-0.00 (-0.01, 0.00), p=0.64	**0.034**
	Year-2	**-1.14 (-1.58, -0.70), p<0.001**		**-0.53 (-0.81, -0.25), p<0.001**		**-0.60 (-0.85, -0.35), p<0.001**		**-0.01 (-0.02, -0.00), p=0.001**	
**Single CMI**	Baseline	0.02 (-0.52, 0.56), p=0.94	**0.014**	0.02 (-0.32, 0.37), p=0.89	0.14	-0.00 (-0.31, 0.30), p=0.99	**0.008**	-0.00 (-0.01, 0.01), p=0.81	0.21
	Year-2	**-0.92 (-1.47, -0.38), p<0.001**		-0.34 (-0.68, 0.01), p=0.05		**-0.59 (-0.89, -0.28), p<0.001**		**-0.01 (-0.01, -0.00), p=0.047**	
**Multiple CMIs**	Baseline	0.07 (-0.54, 0.69), p=0.82	**<0.001**	0.04 (-0.36, 0.43), p=0.86	**0.001**	0.03 (-0.32, 0.39), p=0.85	**0.005**	-0.00 (-0.01, 0.01), p=0.53	0.05
	Year-2	**-1.42 (-2.04, -0.81), p<0.001**		**-0.80 (-1.19, -0.41), p<0.001**		**-0.62 (-0.98, -0.27), p<0.001**		**-0.01 (-0.02, -0.01), p=0.002**	

[a]Brain volumetric parameters were normalized to the intracranial volume and reported as a percentage to account for variations in head size. Model adjusted for age, sex, hypertension, hyperlipidemia, diabetes, smoking status, atrial fibrillation, cortical infarcts, lacunes, cerebral microbleeds, white matter hyperintensity volume, and baseline brain volumes. The results were denoted in bold if reaching statistical significance.

[b]Cortical CMIs were categorized as: 1) absent and present; 2) absent, single (=1), and multiple (≥2). Patients without cortical CMIs served as the reference group.

[c]p value for the interaction between CMI category and time.

Abbreviation: CMI, cerebral microinfarct.

Supplementary Table 3. Synergistic effects of cortical cerebral microinfarcts and brain atrophy on global cognitive function[a]

Categories[b]	Time points	MMSE	MoCA	Global cognition
		β (95%CI), p value	β (95%CI), p value	β (95%CI), p value
**Low brain volume loss + Single CMI**	Baseline	-0.34 (-1.64, 0.97), p=0.61	-0.37 (-1.33, 0.59), p=0.45	-0.32 (-1.31, 0.68), p=0.53
	Year-1	0.03 (-1.27, 1.34), p=0.96	-0.50 (-1.46, 0.46), p=0.30	-0.48 (-1.48, 0.51), p=0.34
	Year-2	-0.38 (-1.68, 0.92), p=0.57	-0.41 (-1.37, 0.55), p=0.40	-0.39 (-1.39, 0.61), p=0.44
	Year-3	-0.16 (-1.48, 1.16), p=0.81	-0.46 (-1.43, 0.51), p=0.35	-0.41 (-1.42, 0.60), p=0.43
	Year-4	0.13 (-1.25, 1.51), p=0.85	-0.44 (-1.44, 0.57), p=0.39	-0.50 (-1.55, 0.55), p=0.35
	Year-5	-0.88 (-2.26, 0.51), p=0.21	-0.66 (-1.66, 0.35), p=0.20	-0.73 (-1.77, 0.32), p=0.17
	p^c	0.50	0.51	0.3
**Low brain volume loss + Multiple CMIs**	Baseline	-0.35 (-1.69, 0.98), p=0.60	-0.16 (-1.14, 0.82), p=0.75	-0.03 (-1.06, 0.99), p=0.95
	Year-1	-0.58 (-1.91, 0.76), p=0.40	-0.40 (-1.39, 0.58), p=0.42	-0.32 (-1.34, 0.70), p=0.54
	Year-2	-0.37 (-1.71, 0.96), p=0.58	-0.51 (-1.49, 0.47), p=0.31	-0.33 (-1.35, 0.69), p=0.53
	Year-3	-1.05 (-2.40, 0.30), p=0.13	-0.62 (-1.61, 0.37), p=0.22	-0.44 (-1.47, 0.59), p=0.40
	Year-4	-1.40 (-2.80, 0.00), p=0.05	-0.64 (-1.67, 0.38), p=0.22	-0.74 (-1.80, 0.33), p=0.17
	Year-5	-1.20 (-2.63, 0.23), p=0.10	-0.91 (-1.95, 0.12), p=0.08	-1.02 (-2.10, 0.06), p=0.07
	p^c	**0.005**	**0.008**	**0.001**
**High brain volume loss + CMI absent**	Baseline	**-0.87 (-1.48, -0.27), p=0.005**	**-0.74 (-1.19, -0.30), p=0.001**	**-0.76 (-1.22, -0.29), p=0.001**
	Year-1	**-1.26 (-1.86, -0.65), p<0.001**	**-1.00 (-1.45, -0.55), p<0.001**	**-0.91 (-1.37, -0.44), p<0.001**
	Year-2	**-1.42 (-2.04, -0.81), p<0.001**	**-1.16 (-1.61, -0.71), p<0.001**	**-1.12 (-1.59, -0.65), p<0.001**
	Year-3	**-1.66 (-2.29, -1.04), p<0.001**	**-1.42 (-1.88, -0.96), p<0.001**	**-1.44 (-1.91, -0.96), p<0.001**
	Year-4	**-1.90 (-2.54, -1.25), p<0.001**	**-1.45 (-1.92, -0.99), p<0.001**	**-1.72 (-2.20, -1.23), p<0.001**
	Year-5	**-2.07 (-2.73, -1.42), p<0.001**	**-1.39 (-1.86, -0.91), p<0.001**	**-1.82 (-2.32, -1.33), p<0.001**
	p^c	**<0.001**	**<0.001**	**<0.001**
**High brain volume loss + Single CMI**	Baseline	**-1.42 (-2.48, -0.37), p=0.008**	**-1.01 (-1.78, -0.23), p=0.011**	**-0.82 (-1.62, -0.01), p=0.046**
	Year-1	**-1.73 (-2.78, -0.67), p=0.001**	**-1.32 (-2.09, -0.54), p<0.001**	**-1.34 (-2.14, -0.53), p=0.001**
	Year-2	**-1.68 (-2.74, -0.61), p=0.002**	**-1.52 (-2.30, -0.74), p<0.001**	**-1.46 (-2.27, -0.65), p<0.001**
	Year-3	**-2.34 (-3.43, -1.24), p<0.001**	**-1.78 (-2.58, -0.98), p<0.001**	**-1.57 (-2.40, -0.74), p<0.001**
	Year-4	**-2.56 (-3.71, -1.41), p<0.001**	**-1.53 (-2.36, -0.70), p<0.001**	**-1.70 (-2.56, -0.83), p<0.001**
	Year-5	**-2.69 (-3.82, -1.56), p<0.001**	**-1.85 (-2.67, -1.03), p<0.001**	**-1.83 (-2.68, -0.97), p<0.001**
	p^c	**<0.001**	**<0.001**	**<0.001**
**High brain volume loss + Multiple CMIs**	Baseline	**-2.07 (-3.36, -0.79), p=0.002**	**-1.38 (-2.32, -0.43), p=0.004**	**-1.91 (-2.89, -0.92), p<0.001**
	Year-1	**-2.44 (-3.73, -1.16), p<0.001**	**-1.62 (-2.57, -0.67), p<0.001**	**-2.14 (-3.13, -1.16), p<0.001**
	Year-2	**-2.67 (-3.95, -1.38), p<0.001**	**-1.93 (-2.88, -0.98), p<0.001**	**-2.64 (-3.63, -1.66), p<0.001**
	Year-3	**-2.97 (-4.29, -1.65), p<0.001**	**-1.94 (-2.91, -0.97), p<0.001**	**-3.07 (-4.07, -2.06), p<0.001**
	Year-4	**-3.09 (-4.48, -1.71), p<0.001**	**-2.14 (-3.15, -1.14), p<0.001**	**-3.16 (-4.20, -2.11), p<0.001**
	Year-5	**-3.53 (-4.96, -2.09), p<0.001**	**-2.27 (-3.31, -1.24), p<0.001**	**-3.13 (-4.21, -2.05), p<0.001**
	p^c	**<0.001**	**<0.001**	**<0.001**

[a]Model adjusted for age, sex, years of education, hypertension, hyperlipidemia, diabetes, smoking status, atrial fibrillation, cortical infarcts, lacunes, cerebral microbleeds, and white matter hyperintensity volume. The results were denoted in bold if reaching statistical significance.
[b]Low and high brain volume loss were determined based on the median split of changes in total brain volume from baseline to year 2. Six interaction groups between brain volume loss and cortical CMI categories were created to examine their combined effects on cognitive decline. Patients with low brain volume loss and no cortical CMIs served as the reference group.
[c]p value for the interaction between each category and time.
Abbreviation: CMI, cerebral microinfarct; MMSE, Mini-Mental State Examination; MoCA, Montreal Cognitive Assessment.

Supplementary Table 4. Synergistic effects of cortical cerebral microinfarcts and brain atrophy on specific cognitive domains[a]

Categories[b]	Time points	Attention	Executive function	Memory	Language	Visuomotor speed	Visuospatial function
		**β (95%CI), p value**	**β (95%CI), p value**	**β (95%CI), p value**	**β (95%CI), p value**	**β (95%CI), p value**	**β (95%CI), p value**
**Low brain volume loss + Single CMI**	Baseline	0.18 (-0.36, 0.73), p=0.51	-0.68 (-1.52, 0.16), p=0.11	-0.17 (-0.82, 0.48), p=0.61	0.18 (-1.85, 2.22), p=0.86	-0.39 (-0.80, 0.02), p=0.06	-0.47 (-1.24, 0.31), p=0.24
	Year-1	0.08 (-0.46, 0.62), p=0.77	-0.90 (-1.73, -0.06), p=0.035	-0.51 (-1.16, 0.13), p=0.12	0.07 (-1.96, 2.10), p=0.95	-0.41 (-0.81, -0.00), p=0.049	-0.38 (-1.15, 0.40), p=0.34
	Year-2	0.04 (-0.50, 0.58), p=0.89	-0.82 (-1.66, 0.01), p=0.05	-0.30 (-0.94, 0.35), p=0.37	0.05 (-1.99, 2.08), p=0.96	-0.35 (-0.76, 0.05), p=0.09	-0.27 (-1.05, 0.51), p=0.49
	Year-3	-0.08 (-0.64, 0.48), p=0.78	-0.71 (-1.56, 0.14), p=0.10	-0.24 (-0.90, 0.41), p=0.47	0.34 (-1.74, 2.41), p=0.75	-0.39 (-0.80, 0.03), p=0.07	-0.63 (-1.43, 0.16), p=0.12
	Year-4	-0.26 (-0.86, 0.33), p=0.39	-1.04 (-1.92, -0.16), p=0.020	-0.52 (-1.20, 0.16), p=0.13	0.65 (-1.56, 2.86), p=0.56	-0.53 (-0.95, -0.10), p=0.015	-0.39 (-1.23, 0.45), p=0.37
	Year-5	-0.32 (-0.92, 0.28), p=0.30	-0.94 (-1.82, -0.06), p=0.037	-0.63 (-1.31, 0.05), p=0.07	0.09 (-2.12, 2.30), p=0.94	-0.49 (-0.92, -0.06), p=0.024	-0.78 (-1.63, 0.06), p=0.07
	p^c	0.013	0.35	0.10	0.75	0.34	0.35
**Low brain volume loss + Multiple CMIs**	Baseline	0.08 (-0.47, 0.64), p=0.77	-0.31 (-1.17, 0.55), p=0.48	-0.22 (-0.88, 0.44), p=0.51	0.44 (-1.64, 2.52), p=0.68	-0.16 (-0.57, 0.26), p=0.46	0.02 (-0.77, 0.81), p=0.96
	Year-1	-0.34 (-0.90, 0.21), p=0.23	-0.57 (-1.43, 0.29), p=0.19	-0.50 (-1.17, 0.16), p=0.14	0.54 (-1.54, 2.62), p=0.61	-0.31 (-0.73, 0.11), p=0.14	-0.16 (-0.95, 0.63), p=0.70
	Year-2	-0.18 (-0.74, 0.37), p=0.52	-0.62 (-1.48, 0.24), p=0.16	-0.56 (-1.22, 0.11), p=0.10	0.66 (-1.42, 2.74), p=0.53	-0.41 (-0.83, 0.00), p=0.05	-0.29 (-1.08, 0.50), p=0.47
	Year-3	-0.25 (-0.81, 0.31), p=0.39	-0.71 (-1.57, 0.16), p=0.11	-0.42 (-1.09, 0.25), p=0.22	0.10 (-2.01, 2.21), p=0.93	-0.51 (-0.93, -0.09), p=0.017	-0.09 (-0.90, 0.71), p=0.82
	Year-4	-0.19 (-0.79, 0.41), p=0.53	-1.03 (-1.93, -0.14), p=0.023	-0.56 (-1.25, 0.13), p=0.11	-0.31 (-2.54, 1.92), p=0.79	-0.50 (-0.94, -0.07), p=0.023	-0.54 (-1.39, 0.31), p=0.22
	Year-5	-0.41 (-1.03, 0.21), p=0.20	-0.67 (-1.58, 0.24), p=0.15	-0.78 (-1.48, -0.07), p=0.030	-1.66 (-3.95, 0.62), p=0.15	-0.39 (-0.83, 0.05), p=0.08	-0.39 (-1.27, 0.48), p=0.38
	p^c	0.14	0.022	0.030	0.018	**0.005**	0.12
**High brain volume loss + CMI absent**	Baseline	-0.17 (-0.42, 0.08), p=0.19	**-0.68 (-1.07, -0.29), p<0.001**	**-0.53 (-0.83, -0.23), p<0.001**	-0.94 (-1.89, 0.01), p=0.05	**-0.34 (-0.53, -0.15), p<0.001**	**-0.55 (-0.91, -0.19), p=0.003**
	Year-1	-0.18 (-0.44, 0.07), p=0.16	**-0.90 (-1.29, -0.51), p<0.001**	**-0.86 (-1.16, -0.56), p<0.001**	-1.01 (-1.96, -0.06), p=0.037	**-0.43 (-0.62, -0.24), p<0.001**	-0.46 (-0.82, -0.10), p=0.013
	Year-2	-0.33 (-0.59, -0.07), p=0.013	**-1.06 (-1.45, -0.67), p<0.001**	**-0.80 (-1.10, -0.50), p<0.001**	**-1.32 (-2.28, -0.36), p=0.007**	**-0.49 (-0.68, -0.30), p<0.001**	**-0.73 (-1.10, -0.36), p<0.001**
	Year-3	**-0.52 (-0.79, -0.26), p<0.001**	**-1.16 (-1.56, -0.76), p<0.001**	**-0.87 (-1.18, -0.56), p<0.001**	**-2.25 (-3.24, -1.27), p<0.001**	**-0.54 (-0.74, -0.35), p<0.001**	**-0.73 (-1.10, -0.35), p<0.001**
	Year-4	**-0.66 (-0.94, -0.38), p<0.001**	**-1.18 (-1.59, -0.77), p<0.001**	**-0.99 (-1.31, -0.68), p<0.001**	**-2.96 (-4.00, -1.93), p<0.001**	**-0.56 (-0.76, -0.36), p<0.001**	**-0.89 (-1.28, -0.49), p<0.001**
	Year-5	**-0.60 (-0.88, -0.31), p<0.001**	**-1.40 (-1.81, -0.98), p<0.001**	**-0.90 (-1.22, -0.58), p<0.001**	**-3.30 (-4.35, -2.25), p<0.001**	**-0.52 (-0.72, -0.32), p<0.001**	**-0.97 (-1.37, -0.57), p<0.001**
	p^c	**<0.001**	**<0.001**	**<0.001**	**<0.001**	**<0.001**	**<0.001**
**High brain volume loss + Single CMI**	Baseline	-0.07 (-0.51, 0.37), p=0.77	-0.81 (-1.49, -0.14), p=0.019	-0.65 (-1.18, -0.13), p=0.014	-1.18 (-2.83, 0.46), p=0.16	-0.29 (-0.61, 0.04), p=0.09	-0.46 (-1.09, 0.16), p=0.15
	Year-1	-0.34 (-0.78, 0.10), p=0.13	**-1.47 (-2.15, -0.79), p<0.001**	**-1.10 (-1.62, -0.58), p<0.001**	-1.31 (-2.96, 0.33), p=0.12	**-0.58 (-0.91, -0.25), p<0.001**	**-0.85 (-1.48, -0.22), p=0.008**
	Year-2	-0.42 (-0.87, 0.03), p=0.07	**-1.67 (-2.35, -0.98), p<0.001**	**-1.02 (-1.55, -0.49), p<0.001**	-1.56 (-3.23, 0.11), p=0.07	**-0.68 (-1.01, -0.35), p<0.001**	**-0.86 (-1.50, -0.22), p=0.008**
	Year-3	-0.58 (-1.05, -0.12), p=0.014	**-1.66 (-2.36, -0.96), p<0.001**	**-1.18 (-1.71, -0.64), p<0.001**	-1.61 (-3.35, 0.12), p=0.07	**-0.71 (-1.05, -0.37), p<0.001**	**-0.92 (-1.58, -0.26), p=0.007**
	Year-4	-0.67 (-1.18, -0.17), p=0.009	**-1.83 (-2.56, -1.10), p<0.001**	**-1.17 (-1.73, -0.60), p<0.001**	-1.69 (-3.55, 0.17), p=0.08	**-0.91 (-1.27, -0.56), p<0.001**	-0.93 (-1.64, -0.22), p=0.010
	Year-5	-0.54 (-1.04, -0.05), p=0.030	**-1.99 (-2.71, -1.27), p<0.001**	**-1.16 (-1.72, -0.61), p<0.001**	-2.23 (-4.04, -0.41), p=0.016	**-0.65 (-1.00, -0.31), p<0.001**	**-1.18 (-1.87, -0.49), p<0.001**
	p^c	**0.002**	**<0.001**	**0.002**	0.15	**<0.001**	0.012
**High brain volume loss + Multiple CMIs**	Baseline	-0.28 (-0.81, 0.26), p=0.31	**-1.34 (-2.17, -0.52), p=0.001**	-0.56 (-1.20, 0.08), p=0.09	**-4.39 (-6.38, -2.40), p<0.001**	-0.46 (-0.86, -0.06), p=0.025	**-1.04 (-1.80, -0.28), p=0.007**
	Year-1	-0.49 (-1.02, 0.04), p=0.07	**-1.85 (-2.68, -1.03), p<0.001**	**-0.96 (-1.60, -0.32), p=0.003**	**-3.82 (-5.82, -1.82), p<0.001**	**-0.67 (-1.07, -0.27), p=0.001**	**-1.28 (-2.04, -0.52), p=0.001**
	Year-2	-0.48 (-1.01, 0.06), p=0.08	**-1.88 (-2.71, -1.05), p<0.001**	**-1.08 (-1.72, -0.44), p<0.001**	**-5.59 (-7.59, -3.59), p<0.001**	**-0.62 (-1.02, -0.22), p=0.002**	**-1.53 (-2.29, -0.76), p<0.001**
	Year-3	**-0.95 (-1.51, -0.40), p<0.001**	**-1.80 (-2.65, -0.96), p<0.001**	**-1.13 (-1.79, -0.48), p<0.001**	**-6.56 (-8.64, -4.49), p<0.001**	**-0.77 (-1.18, -0.36), p<0.001**	**-1.80 (-2.59, -1.01), p<0.001**
	Year-4	**-1.00 (-1.60, -0.40), p=0.001**	**-2.58 (-3.46, -1.70), p<0.001**	**-1.27 (-1.95, -0.59), p<0.001**	**-5.64 (-7.86, -3.42), p<0.001**	**-1.01 (-1.44, -0.59), p<0.001**	**-1.85 (-2.69, -1.00), p<0.001**
	Year-5	-0.83 (-1.47, -0.20), p=0.010	**-2.21 (-3.12, -1.30), p<0.001**	**-1.10 (-1.80, -0.40), p=0.002**	**-6.84 (-9.17, -4.51), p<0.001**	**-0.77 (-1.21, -0.33), p<0.001**	**-1.61 (-2.50, -0.72), p<0.001**
	p^c	**<0.001**	**<0.001**	**<0.001**	**<0.001**	**<0.001**	**0.003**

[a]Model adjusted for age, sex, years of education, hypertension, hyperlipidemia, diabetes, smoking status, atrial fibrillation, cortical infarcts, lacunes, cerebral microbleeds, and white matter hyperintensity volume. The results were denoted in bold if reaching statistical significance. Bonferroni correction was applied to account for multiple comparisons across six cognitive domains, and the threshold for statistical significance was set as p-value of <0.05/6≈0.008.

[b]Low and high brain volume loss were determined based on the median split of changes in total brain volume from baseline to year 2. Six interaction groups between brain volume loss and cortical CMI categories were created to examine their combined effects on cognitive decline. Patients with low brain volume loss and no cortical CMIs served as the reference group.
[c]p value for the interaction between each category and time.
Abbreviation: CMI, cerebral microinfarct.

Supplementary Table 5. Synergistic effects of cortical CMI count, total brain volume change, and time on cognitive function[a]

Outcome	Cortical CMI count × Total brain volume change × Time interaction	
	β (95%CI)	p value
**MMSE**	**-0.0043 (-0.0075, -0.0012)**	**0.007**
**MoCA**	**-0.0031 (-0.0051, -0.0010)**	**0.003**
**Global cognition**	**-0.0036 (-0.0058, -0.0014)**	**0.001**
**Cognition domains[b]**		
**Attention**	-0.0006 (-0.0023, 0.0011)	0.50
**Executive function**	-0.0022 (-0.0041, -0.0003)	0.023
**Memory**	**-0.0022 (-0.0037, -0.0008)**	**0.002**
**Language**	**-0.0085 (-0.0145, -0.0024)**	**0.006**
**Visuomotor speed**	-0.0008 (-0.0017, 0.0001)	0.09
**Visuospatial function**	-0.0006 (-0.0029, 0.0017)	0.60

[a]Model adjusted for age, sex, years of education, hypertension, hyperlipidemia, diabetes, smoking status, atrial fibrillation, cortical infarcts, lacunes, cerebral microbleeds, and white matter hyperintensity volume. Total brain volume change was calculated as the absolute difference in total brain volume between baseline and year 2. The results were denoted in bold if reaching statistical significance.

[b]Bonferroni correction was applied to account for multiple comparisons across six cognitive domains, and the threshold for statistical significance was set as p-value of $<0.05/6\approx0.008$.

Abbreviation: CMI, cerebral microinfarct; MMSE, Mini-Mental State Examination; MoCA, Montreal Cognitive Assessment.

Supplementary Table 6. Association between cortical cerebral microinfarcts and brain volumetric parameters after excluding patients with large cortical infarcts[a]

Categories[b]	Time points	Total brain volume, %		Gray matter volume, %		White matter volume, %		Total hippocampal volume, %	
		**β (95%CI), p value**	**p[c]**	**β (95%CI), p value**	**p[c]**	**β (95%CI), p value**	**p[c]**	**β (95%CI), p value**	**p[c]**
**CMI present**	Baseline	-0.13 (-1.39, 1.12), p=0.84	**<0.001**	-0.17 (-0.96, 0.62), p=0.67	**0.009**	0.04 (-0.63, 0.70), p=0.91	**0.004**	0.00 (-0.02, 0.02), p=0.83	0.06
	Year-2	**-1.31 (-2.57, -0.06), p=0.040**		-0.76 (-1.56, 0.03), p=0.06		-0.55 (-1.21, 0.12), p=0.11		-0.01 (-0.03, 0.01), p=0.53	
**Single CMI**	Baseline	0.03 (-1.41, 1.47), p=0.97	0.06	-0.13 (-1.04, 0.78), p=0.78	0.19	0.16 (-0.61, 0.92), p=0.69	0.06	0.01 (-0.02, 0.03), p=0.66	0.48
	Year-2	-0.76 (-2.19, 0.68), p=0.30		-0.47 (-1.38, 0.44), p=0.31		-0.28 (-1.04, 0.48), p=0.47		0.00 (-0.02, 0.02), p=0.89	
**Multiple CMIs**	Baseline	-0.54 (-2.65, 1.58), p=0.62	**<0.001**	-0.28 (-1.62, 1.05), p=0.68	**0.002**	-0.26 (-1.38, 0.87), p=0.66	**0.007**	-0.01 (-0.04, 0.03), p=0.75	**0.009**
	Year-2	**-2.64 (-4.76, -0.53), p=0.014**		**-1.46 (-2.79, -0.12), p=0.033**		**-1.18 (-2.30, -0.06), p=0.039**		-0.03 (-0.06, 0.01), p=0.14	

[a]Brain volumetric parameters were normalized to the intracranial volume and reported as a percentage to account for variations in head size. Model adjusted for age, sex, hypertension, hyperlipidemia, diabetes, smoking status, atrial fibrillation, lacunes, cerebral microbleeds, and white matter hyperintensity volume. The results were denoted in bold if reaching statistical significance.

[b]Cortical CMIs were categorized as: 1) absent and present; 2) absent, single (=1), and multiple (≥2). Patients without cortical CMIs served as the reference group.

[c]p value for the interaction between CMI category and time.

Abbreviation: CMI, cerebral microinfarct.

Supplementary Table 7. Synergistic effects of cortical cerebral microinfarcts and brain atrophy on global cognitive function after excluding patients with large cortical infarcts[a]

Categories[b]	Time points	MMSE	MoCA	Global cognition
		**β (95%CI), p value**	**β (95%CI), p value**	**β (95%CI), p value**
**Low brain volume loss + Single CMI**	Baseline	-1.03 (-2.46, 0.40), p=0.16	-0.89 (-1.95, 0.16), p=0.10	-0.58 (-1.66, 0.50), p=0.29
	Year-1	-0.60 (-2.03, 0.83), p=0.41	-0.96 (-2.01, 0.10), p=0.08	-0.82 (-1.90, 0.27), p=0.14
	Year-2	-0.89 (-2.32, 0.54), p=0.22	-0.87 (-1.92, 0.19), p=0.11	-0.81 (-1.90, 0.27), p=0.14
	Year-3	-0.77 (-2.23, 0.68), p=0.30	-1.02 (-2.09, 0.05), p=0.06	-0.82 (-1.93, 0.28), p=0.14
	Year-4	-0.31 (-1.83, 1.21), p=0.69	-0.83 (-1.94, 0.28), p=0.14	-0.71 (-1.85, 0.43), p=0.22
	Year-5	-1.45 (-2.97, 0.07), p=0.06	**-1.27 (-2.38, -0.16), p=0.025**	**-1.23 (-2.38, -0.09), p=0.034**
	p[c]	0.83	0.46	0.15
**Low brain volume loss + Multiple CMIs**	Baseline	-0.25 (-2.06, 1.56), p=0.79	-0.14 (-1.47, 1.20), p=0.84	0.02 (-1.35, 1.40), p=0.97
	Year-1	-0.97 (-2.78, 0.84), p=0.29	-0.55 (-1.88, 0.79), p=0.42	-0.53 (-1.90, 0.84), p=0.45
	Year-2	-0.37 (-2.18, 1.44), p=0.69	-0.62 (-1.95, 0.72), p=0.37	-0.37 (-1.75, 1.00), p=0.59
	Year-3	-1.50 (-3.33, 0.33), p=0.11	-0.93 (-2.28, 0.42), p=0.18	-0.65 (-2.03, 0.74), p=0.36
	Year-4	-1.08 (-3.03, 0.87), p=0.28	-0.46 (-1.88, 0.97), p=0.53	-0.76 (-2.22, 0.70), p=0.31
	Year-5	-1.18 (-3.13, 0.77), p=0.24	-0.85 (-2.27, 0.58), p=0.24	-0.53 (-1.99, 0.93), p=0.48
	p[c]	0.07	0.09	0.11
**High brain volume loss + CMI absent**	Baseline	**-0.95 (-1.58, -0.32), p=0.003**	**-0.77 (-1.24, -0.31), p=0.001**	**-0.77 (-1.24, -0.29), p=0.002**
	Year-1	**-1.29 (-1.92, -0.66), p<0.001**	**-1.05 (-1.52, -0.59), p<0.001**	**-0.92 (-1.40, -0.44), p<0.001**
	Year-2	**-1.61 (-2.24, -0.97), p<0.001**	**-1.24 (-1.71, -0.77), p<0.001**	**-1.16 (-1.64, -0.68), p<0.001**
	Year-3	**-1.77 (-2.41, -1.13), p<0.001**	**-1.49 (-1.96, -1.01), p<0.001**	**-1.48 (-1.96, -0.99), p<0.001**
	Year-4	**-2.00 (-2.66, -1.34), p<0.001**	**-1.51 (-1.99, -1.02), p<0.001**	**-1.73 (-2.23, -1.23), p<0.001**
	Year-5	**-2.19 (-2.86, -1.52), p<0.001**	**-1.43 (-1.92, -0.94), p<0.001**	**-1.83 (-2.33, -1.32), p<0.001**
	p[c]	**<0.001**	**<0.001**	**<0.001**
**High brain volume loss + Single CMI**	Baseline	**-1.84 (-2.98, -0.70), p=0.002**	**-1.34 (-2.18, -0.50), p=0.002**	**-1.11 (-1.97, -0.25), p=0.012**
	Year-1	**-2.13 (-3.27, -0.99), p<0.001**	**-1.70 (-2.54, -0.86), p<0.001**	**-1.72 (-2.59, -0.86), p<0.001**
	Year-2	**-2.14 (-3.29, -0.99), p<0.001**	**-1.86 (-2.70, -1.01), p<0.001**	**-1.81 (-2.68, -0.95), p<0.001**
	Year-3	**-2.91 (-4.09, -1.74), p<0.001**	**-2.25 (-3.12, -1.39), p<0.001**	**-1.98 (-2.87, -1.09), p<0.001**
	Year-4	**-3.06 (-4.28, -1.85), p<0.001**	**-1.95 (-2.84, -1.06), p<0.001**	**-2.11 (-3.02, -1.19), p<0.001**
	Year-5	**-3.28 (-4.48, -2.07), p<0.001**	**-2.34 (-3.22, -1.46), p<0.001**	**-2.26 (-3.17, -1.36), p<0.001**
	p[c]	**<0.001**	**<0.001**	**<0.001**
**High brain volume loss + Multiple CMIs**	Baseline	**-2.49 (-4.38, -0.60), p=0.010**	**-1.71 (-3.10, -0.31), p=0.016**	**-1.53 (-2.97, -0.10), p=0.036**
	Year-1	**-2.36 (-4.27, -0.45), p=0.015**	-1.40 (-2.81, 0.01), p=0.05	**-1.53 (-2.98, -0.09), p=0.038**
	Year-2	**-2.95 (-4.84, -1.06), p=0.002**	**-1.87 (-3.26, -0.47), p=0.009**	**-2.61 (-4.05, -1.18), p<0.001**
	Year-3	**-3.84 (-5.78, -1.91), p<0.001**	**-1.95 (-3.37, -0.52), p=0.007**	**-3.66 (-5.12, -2.20), p<0.001**
	Year-4	**-4.07 (-6.08, -2.06), p<0.001**	**-2.36 (-3.83, -0.89), p=0.002**	**-3.40 (-4.91, -1.89), p<0.001**
	Year-5	**-4.66 (-6.81, -2.52), p<0.001**	**-2.59 (-4.14, -1.03), p=0.001**	**-4.40 (-6.00, -2.81), p<0.001**
	p[c]	**<0.001**	**0.023**	**<0.001**

[a]Model adjusted for age, sex, education, hypertension, hyperlipidemia, diabetes, smoking status, atrial fibrillation, lacunes, cerebral microbleeds, and white matter hyperintensity volume. The results were denoted in bold if reaching statistical significance.
[b]Low and high brain volume loss were determined based on the median split of changes in total brain volume from baseline to year 2. Six interaction groups between brain volume loss and cortical CMI categories were created to examine their combined effects on cognitive decline. Patients with low brain volume loss and no cortical CMIs served as the reference group.
[c]p value for the interaction between each category and time.
Abbreviation: CMI, cerebral microinfarct; MMSE, Mini-Mental State Examination; MoCA, Montreal Cognitive Assessment.

Supplementary Table 8. Synergistic effects of cortical cerebral microinfarcts and brain atrophy on specific cognitive domains after excluding patients with large cortical infarcts[a]

Categories[b]	Time points	Attention	Executive function	Memory	Language	Visuomotor speed	Visuospatial function
		**β (95%CI), p value**	**β (95%CI), p value**	**β (95%CI), p value**	**β (95%CI), p value**	**β (95%CI), p value**	**β (95%CI), p value**
**Low brain volume loss + Single CMI**	Baseline	0.09 (-0.51, 0.68), p=0.78	-0.94 (-1.87, -0.02), p=0.046	-0.39 (-1.12, 0.33), p=0.29	-0.07 (-2.18, 2.04), p=0.95	-0.46 (-0.91, -0.01), p=0.043	-0.67 (-1.52, 0.18), p=0.12
	Year-1	-0.03 (-0.63, 0.56), p=0.91	-1.12 (-2.05, -0.20), p=0.018	-0.64 (-1.36, 0.09), p=0.09	-0.51 (-2.62, 1.60), p=0.64	-0.52 (-0.97, -0.07), p=0.024	-0.65 (-1.50, 0.20), p=0.14
	Year-2	-0.02 (-0.61, 0.58), p=0.95	-1.10 (-2.03, -0.17), p=0.020	-0.50 (-1.23, 0.22), p=0.18	-0.61 (-2.72, 1.51), p=0.57	-0.51 (-0.96, -0.06), p=0.027	-0.71 (-1.56, 0.14), p=0.10
	Year-3	-0.12 (-0.73, 0.49), p=0.70	-1.06 (-2.01, -0.12), p=0.027	-0.52 (-1.26, 0.21), p=0.16	-0.23 (-2.40, 1.94), p=0.83	-0.56 (-1.02, -0.10), p=0.016	-0.97 (-1.84, -0.10), p=0.030
	Year-4	-0.26 (-0.91, 0.40), p=0.44	-1.29 (-2.27, -0.31), p=0.010	-0.72 (-1.48, 0.05), p=0.07	0.24 (-2.08, 2.56), p=0.84	-0.62 (-1.09, -0.14), p=0.011	-0.34 (-1.27, 0.59), p=0.47
	Year-5	-0.50 (-1.16, 0.16), p=0.14	**-1.46 (-2.44, -0.48), p=0.004**	-0.91 (-1.68, -0.15), p=0.019	-0.45 (-2.77, 1.87), p=0.70	**-0.70 (-1.18, -0.22), p=0.004**	-1.20 (-2.14, -0.27), p=0.011
	p[c]	0.027	0.12	0.06	0.94	0.10	0.32
**Low brain volume loss + Multiple CMIs**	Baseline	0.44 (-0.32, 1.19), p=0.26	-0.28 (-1.45, 0.90), p=0.65	-0.32 (-1.24, 0.60), p=0.49	0.49 (-2.18, 3.17), p=0.72	-0.08 (-0.65, 0.49), p=0.78	-0.16 (-1.24, 0.92), p=0.78
	Year-1	-0.31 (-1.06, 0.45), p=0.43	-0.81 (-1.98, 0.37), p=0.18	-0.60 (-1.52, 0.32), p=0.20	0.04 (-2.64, 2.72), p=0.98	-0.23 (-0.80, 0.34), p=0.44	-0.34 (-1.42, 0.74), p=0.54
	Year-2	0.04 (-0.72, 0.79), p=0.93	-0.41 (-1.58, 0.77), p=0.50	-0.84 (-1.76, 0.08), p=0.07	0.64 (-2.04, 3.32), p=0.64	-0.31 (-0.88, 0.26), p=0.28	-0.70 (-1.78, 0.38), p=0.20
	Year-3	-0.20 (-0.97, 0.57), p=0.61	-0.79 (-1.97, 0.39), p=0.19	-0.38 (-1.30, 0.55), p=0.43	-0.32 (-3.04, 2.40), p=0.82	-0.47 (-1.05, 0.10), p=0.11	-0.58 (-1.67, 0.52), p=0.30
	Year-4	0.01 (-0.84, 0.86), p=0.98	-1.34 (-2.59, -0.09), p=0.036	-0.85 (-1.83, 0.13), p=0.09	0.89 (-2.10, 3.88), p=0.56	-0.60 (-1.21, 0.00), p=0.05	-1.31 (-2.51, -0.11), p=0.032
	Year-5	-0.01 (-0.86, 0.84), p=0.98	-0.77 (-2.03, 0.48), p=0.23	-1.05 (-2.03, -0.07), p=0.035	0.39 (-2.60, 3.38), p=0.80	-0.44 (-1.05, 0.16), p=0.15	-0.32 (-1.53, 0.88), p=0.60
	p[c]	0.29	0.049	0.05	0.98	**0.003**	0.13
**High brain volume loss + CMI absent**	Baseline	-0.20 (-0.46, 0.07), p=0.14	**-0.68 (-1.08, -0.27), p=0.001**	**-0.56 (-0.88, -0.24), p<0.001**	-0.90 (-1.83, 0.03), p=0.06	**-0.34 (-0.54, -0.14), p<0.001**	**-0.56 (-0.94, -0.19), p=0.003**
	Year-1	-0.22 (-0.48, 0.04), p=0.10	**-0.91 (-1.32, -0.50), p<0.001**	**-0.89 (-1.21, -0.57), p<0.001**	-0.95 (-1.88, -0.02), p=0.045	**-0.44 (-0.64, -0.24), p<0.001**	-0.47 (-0.85, -0.10), p=0.014
	Year-2	-0.34 (-0.61, -0.08), p=0.012	**-1.06 (-1.47, -0.65), p<0.001**	**-0.84 (-1.16, -0.52), p<0.001**	**-1.42 (-2.36, -0.48), p=0.003**	**-0.51 (-0.71, -0.31), p<0.001**	**-0.73 (-1.11, -0.35), p<0.001**
	Year-3	**-0.55 (-0.82, -0.28), p<0.001**	**-1.20 (-1.61, -0.78), p<0.001**	**-0.90 (-1.22, -0.57), p<0.001**	**-2.26 (-3.23, -1.30), p<0.001**	**-0.56 (-0.76, -0.35), p<0.001**	**-0.77 (-1.16, -0.38), p<0.001**
	Year-4	**-0.71 (-0.99, -0.42), p<0.001**	**-1.22 (-1.64, -0.79), p<0.001**	**-1.03 (-1.36, -0.70), p<0.001**	**-2.90 (-3.91, -1.89), p<0.001**	**-0.57 (-0.78, -0.36), p<0.001**	**-0.89 (-1.29, -0.48), p<0.001**
	Year-5	**-0.63 (-0.93, -0.34), p<0.001**	**-1.44 (-1.88, -1.01), p<0.001**	**-0.93 (-1.27, -0.60), p<0.001**	**-3.13 (-4.15, -2.10), p<0.001**	**-0.52 (-0.73, -0.31), p<0.001**	**-1.02 (-1.43, -0.61), p<0.001**
	p[c]	**<0.001**	**<0.001**	**<0.001**	**<0.001**	**<0.001**	**<0.001**
**High brain volume loss + Single CMI**	Baseline	-0.17 (-0.65, 0.30), p=0.47	**-1.11 (-1.85, -0.38), p=0.003**	**-0.86 (-1.44, -0.28), p=0.004**	-1.43 (-3.12, 0.26), p=0.10	-0.45 (-0.81, -0.09), p=0.014	-0.67 (-1.35, 0.01), p=0.05
	Year-1	-0.49 (-0.97, -0.02), p=0.043	**-1.87 (-2.61, -1.14), p<0.001**	**-1.27 (-1.85, -0.69), p<0.001**	-1.82 (-3.51, -0.13), p=0.034	**-0.74 (-1.10, -0.38), p<0.001**	**-1.09 (-1.77, -0.41), p=0.002**
	Year-2	-0.54 (-1.02, -0.06), p=0.026	**-2.04 (-2.78, -1.30), p<0.001**	**-1.18 (-1.76, -0.60), p<0.001**	-2.00 (-3.71, -0.29), p=0.022	**-0.85 (-1.21, -0.49), p<0.001**	**-1.08 (-1.77, -0.39), p=0.002**
	Year-3	-0.61 (-1.11, -0.11), p=0.017	**-2.09 (-2.85, -1.33), p<0.001**	**-1.33 (-1.93, -0.74), p<0.001**	-2.22 (-3.98, -0.46), p=0.014	**-0.88 (-1.25, -0.51), p<0.001**	**-1.22 (-1.93, -0.52), p<0.001**
	Year-4	**-0.78 (-1.31, -0.25), p=0.004**	**-2.26 (-3.05, -1.48), p<0.001**	**-1.37 (-1.98, -0.76), p<0.001**	-2.18 (-4.05, -0.32), p=0.022	**-1.11 (-1.49, -0.73), p<0.001**	**-1.22 (-1.97, -0.47), p=0.001**
	Year-5	**-0.72 (-1.24, -0.20), p=0.007**	**-2.39 (-3.16, -1.61), p<0.001**	**-1.34 (-1.94, -0.73), p<0.001**	**-2.73 (-4.57, -0.90), p=0.003**	**-0.83 (-1.21, -0.45), p<0.001**	**-1.55 (-2.29, -0.82), p<0.001**
	p[c]	**0.003**	**<0.001**	**0.006**	0.07	**<0.001**	**0.003**
**High brain volume loss + Multiple CMIs**	Baseline	-0.02 (-0.81, 0.76), p=0.95	-1.56 (-2.78, -0.33), p=0.013	-0.75 (-1.71, 0.21), p=0.13	-3.11 (-5.90, -0.31), p=0.030	-0.37 (-0.96, 0.23), p=0.23	-0.68 (-1.81, 0.45), p=0.24
	Year-1	-0.55 (-1.35, 0.25), p=0.18	**-1.70 (-2.94, -0.47), p=0.007**	-1.09 (-2.06, -0.12), p=0.027	-2.00 (-4.84, 0.84), p=0.17	-0.67 (-1.27, -0.07), p=0.030	-0.53 (-1.68, 0.61), p=0.36
	Year-2	-0.54 (-1.33, 0.25), p=0.18	**-1.89 (-3.12, -0.67), p=0.002**	-1.29 (-2.25, -0.33), p=0.009	**-5.29 (-8.09, -2.49), p<0.001**	-0.54 (-1.13, 0.06), p=0.08	-1.50 (-2.63, -0.37), p=0.009
	Year-3	**-1.24 (-2.06, -0.42), p=0.003**	**-1.94 (-3.20, -0.69), p=0.002**	-1.19 (-2.17, -0.21), p=0.017	**-8.93 (-11.84, -6.03), p<0.001**	**-0.87 (-1.48, -0.26), p=0.005**	-1.40 (-2.57, -0.23), p=0.019
	Year-4	**-1.48 (-2.36, -0.61), p<0.001**	**-2.58 (-3.88, -1.29), p<0.001**	**-1.58 (-2.59, -0.57), p=0.002**	**-6.51 (-9.58, -3.45), p<0.001**	**-1.00 (-1.63, -0.38), p=0.002**	-1.20 (-2.43, 0.03), p=0.06
	Year-5	**-1.88 (-2.84, -0.92), p<0.001**	-1.81 (-3.18, -0.44), p=0.010	**-1.48 (-2.55, -0.41), p=0.007**	**-11.38 (-14.74, -8.03), p<0.001**	-0.64 (-1.30, 0.03), p=0.06	-1.61 (-2.95, -0.26), p=0.019
	p[c]	**<0.001**	0.06	0.010	**<0.001**	**0.008**	0.023

[a]Model adjusted for age, sex, education, hypertension, hyperlipidemia, diabetes, smoking status, atrial fibrillation, lacunes, cerebral microbleeds, and white matter hyperintensity volume. The results were denoted in bold if reaching statistical significance. Bonferroni correction was applied to account for multiple comparisons across six cognitive domains, and the threshold for statistical significance was set as p-value of $<0.05/6\approx0.008$.

[b]Low and high brain volume loss were determined based on the median split of changes in total brain volume from baseline to year 2. Six interaction groups between brain volume loss and cortical CMI categories were created to examine their combined effects on cognitive decline. Patients with low brain volume loss and no cortical CMIs served as the reference group.
[c]p value for the interaction between each category and time.
Abbreviation: CMI, cerebral microinfarct.
